# Supplementary material for: Novel hybrid silicon-lipid nanoparticles deliver a siRNA to cure autosomal dominant osteopetrosis in mice. Implications for gene therapy in humans
Source: Mol Ther Nucleic Acids. 2023 Aug 19;33:925–37. doi: 10.1016/j.omtn.2023.08.020 (PMC10480457; doi:10.1016/j.omtn.2023.08.020)
Supplement: Document S1. Figures S1 and Tables S1–S8 [file mmc1.pdf]

OMTN, Volume *33*

Supplemental information

Novel hybrid silicon-lipid nanoparticles deliver a siRNA to cure autosomal dominant osteopetrosis in mice. Implications for gene therapy in humans

Antonio Maurizi, Piergiorgio Patrizii, Anna Teti, Flavia Maria Sutera, Paulina Baran-Rachwalska, Chris Burns, Uttom Nandi, Michael Welsh, Nissim Torabi-Pour, Ashkan Dehsorkhi, and Suzanne Saffie-Siebert

Supplemental Information

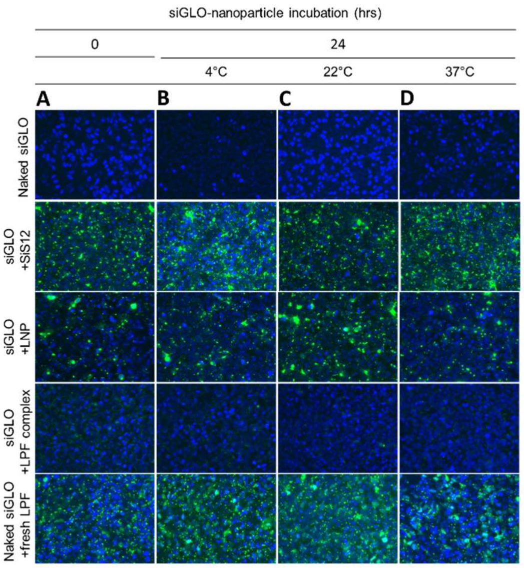

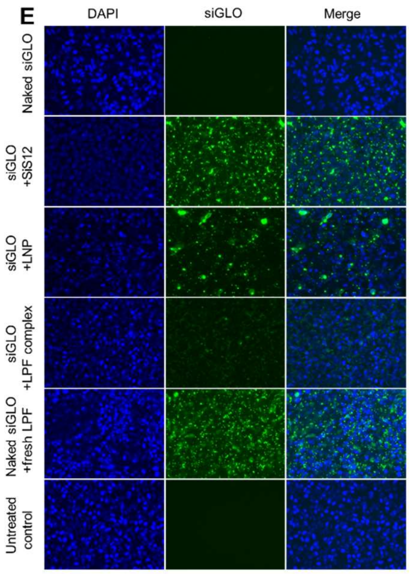

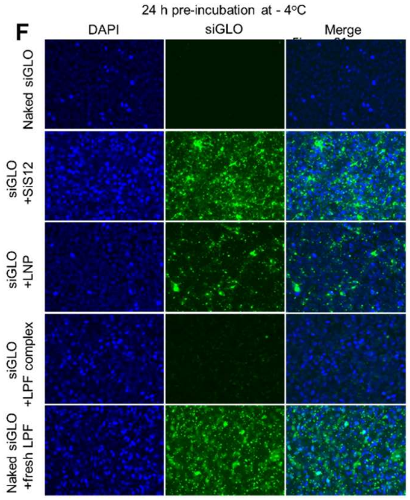

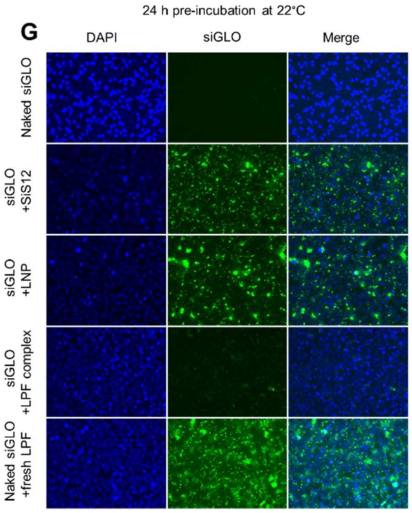

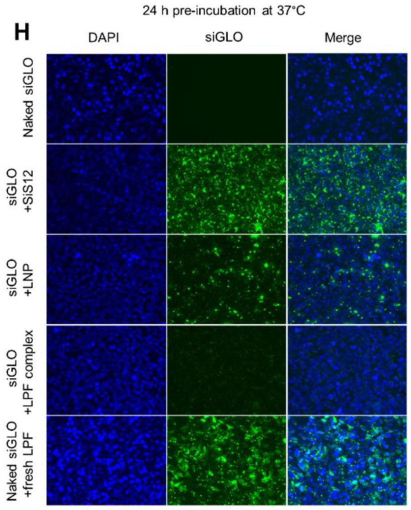

Figure S1. Assessment of cellular uptake of siGLO. Green-fluorescent siGLO (50 pmol) was complexed with SiS12 sshLNP prototype, lipid nanoparticles (LPN) or Lipofectamine (LPF) reagent and administered to AD293 cells **(A)** freshly prepared (time 0) or after incubation for 24 hrs at **(B)** 4°C, **(C)** 22°C or **(D)** 37°C. Treated AD293 cells were then cultured for 24 hrs at 37°C, in a humified atmosphere of 5% CO_2. Naked siGLO was subjected to the same treatment as control. For positive transfection control, a freshly prepared Lipofectamine (LPF) complex with siGLO (siGLO+fresh LPF) was used. At the end of incubation, cells were washed, fixed with 4% paraformaldehyde, stained with DAPI nuclear dye (blue) and imaged by fluoresce microscopy under 200x magnification. Pictures are representative of 3 experiments with n=6 replicates per condition and show merged images. **(E-H)** Separate blue and green images alongside merge images.

Table S1. Composition of all sshLNP prototypes tested.

Prototypes	Lipids				Silicon	Glycine	Trehalose	PoLyLysine	PEI (5)
	DOTAP (1)	DOPE (2)	mPEG2000-DSPE (3)	DPPC (4)					
**SiS 1**	7.25	7.30	1.45	NA(6)	2	1	2	NA	NA
**SiS 2**	7.25	7.30	1.45	NA	1	0.5	1	NA	NA
**SiS 3**	7.45	3.55	1.45	3.55	1	0.5	1	NA	NA
**SiS A**	NA	11.25	4.75	NA	1	0.5	1	3.2	NA
**SiS B**	7.25	7.30	1.45	NA	1	0.5	1	NA	NA
**SiS C**	NA	11.25	4.75	NA	1	0.5	1	NA	3.2
**SiS D**	7.25	7.30	1.45	NA	1	0.5	1	NA	0.8

(1)DOTAP: 1,2-Dioleoyl-3-trimethylammonium propane
(2)DOPE: Dioleoyl-phosphatidyl-ethanolamine
(3)mPEG2000-DSPE: N-(carbonyl-methoxypolyethylene glycol-2000)-1,2-distearoyl-sn-glycero-3-phosphoethanolamine, sodium salt
(4)DPPC: Dipalmitoyl-phosphatidyl-choline
(5)PEI: Polyethylenimine
(6)NA: Not applicable

Table S2. Primer sequences and PCR conditions.

Primer pairs	Sequence	PCR conditions
		95° 10'<br>40 cycles 95° 30'', 60° 30'', 72° 30''
Clcn7^{G213R}	Fw: CAAGTGCTTCCTCAATA<br>Rv: GCCCTCTTCCAAGCTAAACA	
Il-6	Fw: GTTCTCTGGGAAATCGTGGA<br>Rv: GGAAATTGGGGTAGGAAGGA	
Il-1β	Fw: GCCCATCCTCTGTGACTCAT<br>Rv: AGGCCACAGGTATTTTGTCG	
Tnf-α	Fw: CTCCCTTTGCAGAACTCAGG<br>Rv: AGCCCCCAGTCTGTATCCTT	
Ifn-γ	Fw: TTTGAGGTCAACAACCCACA<br>Rv: CGCAATCACAGTCTTGGCTA	
Gapdh	Fw: TGGCAAAGTGGAGATTGTTGC<br>Rv: AAGATGGTGATGGGCTTCCCG	
Clcn3	Fw: GGTCAGGATGGCTTGTTGTT<br>Rv: ACTCTGCCCATGTTTTCCAC	
Clcn5	Fw: GCCTTTGAAGCCACAGAAAG<br>Rv: CTAGCCACCGACTTTTGCTC	

Table S3. Statistical comparison. Data reported in Figure 1 related to the effects induced by the siRNA-SiS12 and siRNA-SiS13 formulations on serum CTX levels (Figure 1B,C) and *Clcn7*^{G213R} mRNA expression in femurs (Figure 1D,E) and PBMCs (Figure 1F,G) of treated ADO2 mice were subjected to statistical comparison.

	**CTX Fold changes**			**Clcn7G213R mRNA (Rel. Expression)**		
	**SiS12-siRNA**	**Sis13+siRNA**	**p-value**	**-**		**p-value**
**Serum**	1.14±0.15	1.18±0,12	ns[(1)]	-		
**Femurs**	-		-	1.06±0.38	1.00±0.48	ns
**PBMCs**	-		-	0.61±0.18	1.00±0.26	0.024

Statistics: Data are the mean ± SD of n=5 mice per group. Student's *t* test. [(1)]ns: statistically not significant.

Table S4. siGLO transfection of AD293 cells.

	siGLO fluorescence							
Samples	Freshly prepared		24 h pre-incubation at 4ºC		24 h pre-incubation at RT[1]		24 h pre-incubation at 37ºC	
	Mean±SD	P value	Mean±SD	P value	Mean±SD	P value	Mean±SD	P value
Naked siGLO	31.4±149.0	<0.0001	27.1±50.7	<0.0001	64.5±180.7	<0.0001	122.2±251.8	<0.0001
siGLO+SiS12	5984.8±475.6	<0.0001	5877.8±492.2	<0.0001	5083.6±1537.0	<0.01	5036.0±1513.2	<0.0001
siGLO+LNP	6254.8±847.6	<0.0001	8315.2±1128.8	<0.0001	5827.4±1740.3	<0.0001	3545.6±170.8	<0.05
siGLO+LPF complex	1611.8±374.6	<0.0001	1673.8±250.6	<0.0001	2232.7±437.9	<0.0001	1725.8±332.5	<0.0001
siGLO+fresh LPF[2]	4121.8±183.5	ns[4]	5968.0±1845.3	ns	6210.7±884.2	ns	4121.8±183.5	ns
Untreated[3]	0.0±79.3	<0.0001	0.0±90.8	<0.0001	0.0±73.7	<0.0001	0.0±49.5	<0.0001
	Relative siGLO/Hoechst fluorescence							
Naked siGLO	0.003±0.003	<0.0001	0.002±0.004	<0.0001	0.003±0.009	<0.0001	0.009±0.018	<0.0001
siGLO+SiS12	0.367±0.367	<0.0001	0.370±0.065	ns	0.480±0.154	<0.0001	0.454±0.089	<0.0001
siGLO+LNP	0.214±0.214	<0.0001	0.622±0.091	<0.0001	0.683±0.190	<0.0001	0.216±0.016	<0.0001
siGLO+LPF complex	0.094±0.094	<0.0001	0.114±0.023	<0.0001	0.119±0.029	<0.0001	0.117±0.011	<0.0001
siGLO+fresh LPF[2]	0.524±0.524	ns	0.405±0.029	ns	0.313±0.030	ns	0.524±0.085	ns
Untreated[3]	0.000±0.000	<0.0001	-0.001±0.006	<0.0001	0.000±0.007	<0.0001	0.000±0.003	<0.0001
Naked siGLO	0.003±0.003	<0.0001	0.002±0.004	<0.0001	0.003±0.009	<0.0001	0.009±0.018	<0.0001

[1]RT: Room temperature; [2]Positive control; [3]Negative control; [4]ns: Not significant

Note: Ordinary One Way ANOVA with Tukey's multiple comparison test – tabular significance reported for all groups with respect to siGLO+LPF fresh, per each single storage condition tested.

For siGLO fluorescence: On freshly prepared samples: siGLO+SiS12 vs siGLO+LNP (p=0.9980) and Naked siGLO vs Untreated (p=0.2447) were not significant. On 24h pre-incubation at 4ºC: Naked siGLO vs Untreated (p=0.9988) was not significant. On 24h pre-incubation at RT: Naked siGLO vs Untreated (p=0.9988) was not significant; siGLO+SiS12 vs siGLO+LNP (p=0.1140) and siGLO+LNP vs. siGLO+fresh LPF (p=0.7706) were not significant. On 24h pre-incubation at 37 ºC: Naked siGLO vs Untreated (p=0.9846) was not significant.

For Relative siGLO/Hoechst fluorescence: On freshly prepared samples: siGLO+SiS12 vs siGLO+LNP (p=0.1099) and Naked siGLO vs Untreated (p=0.9999) were not significant. On 24h pre-incubation at 4ºC: Naked siGLO vs Untreated (p=0.9999) was not significant. On 24h pre-incubation at RT: Naked siGLO vs Untreated (p=0.9988) was not significant; naked siGLO vs. siGLO+LPF complex reported significance (p=0.0013); siGLO+LPF complex vs. untreated reported significance (p<0.001). On 24h pre-incubation at 37ºC: Naked siGLO vs Untreated (p=0.9909) was not significant.

Table S5. Percent of organ SiS12-18:1PE-CF10%+siRNA uptake in vivo.

	Liver	Bones	Kidneys	Lungs	Spleen	Serum
**IP injection**	**% SiS12-18:1PE-CF10%+siRNA RFU (485/Em5-20)**					
**6h**	0	0	1.32	0	1.7	96.98
**24h**	51.12	15.11	13.78	8.51	5.79	5.69
**48h**	56.73	13.52	15.15	7.73	6.87	0
**SC injection**	**% SiS12-18:1PE-CF10%+siRNA RFU (485/Em5-20)**					
**6h**	0	0	0	0	0	100
**24h**	48.02	17.65	16.6	7.89	7.03	2.82
**48h**	47.55	19.45	12.3	14.02	6.67	0

Table S6. Schematic representation of *in vivo* long-term experiment

**Treatment**	**Dose (mg/Kg)**	**N. of mice**
SiS12 combined with naked siRNA *CLCN7*^{G215R}	4	5
SiS12 combined with naked siRNA *CLCN7*^{G215R}	2	5
SiS12 combined with naked siRNA *CLCN7*^{G215R}	0.2	5
SiS12 Empty	SiS12 only	5
Naked siRNA *CLCN7*^{G215R} alone	4	5
Positive control, generated by combining in-vivoJetPEI® with dAdT siRNA *CLCN7*^{G215R} (1)	4	5
Untreated wildtype mice injected with saline (NaCl 0.9%)	0.9% NaCl	5

Table S7. ADO2 siRNA sequences

**Length**	**Strand**	**siRNA ADO2 with dTdT overhang**	**siRNA ADO2 with dAdT overhang**
21-mers	Sense	5' AACAGGGUGAAGAUCCCCCdTdT 3'	5' AACAGGGUGAAGAUCCCCCdAdT 3’
21-mers	Antisense	5'GGGGGAUCUUCACCCUGUUdTdT 3'	5' GGGGGAUCUUCACCCUGUUdAdT 3'

Table S8. Animal methods and Animal Research Reporting of In Vivo Experiments (ARRIVE) compliance.

Ethical statement	All the in vivo experiments were conducted in agreement with the national and international guidelines and policies (European Economic Community Council Directive 86/609, OJ L 358, 1, December 12, 1987; Italian Legislative Decree 4.03.2014, n.26, ***Gazzetta Ufficiale della Repubblica Italiana*** no. 61, March 4, 2014) and were approved by the Italian Ministry of Health (approval n. 112/2020-PR).
Study design	Intraperitoneal (i.p.) injections of SiSaf-SiS sshLNP + *Clcn7*^{G213R}-siRNA or SiSaf-SiS sshLNP Empty or Saline in vivo:<br>a. Experimental groups: WT Saline (5 mice), ADO2 SiSaf-SiS sshLNP + *Clcn7*^{G213R}-siRNA (5 mice), ADO2 SiSaf-SiS sshLNP Empty (5 mice).<br>b. Experimental units:<br>i. Young mice (10 days old): groups of animals from different litters.<br>c. Samples that were not analyzed were stored and will be used for other projects, according to the 3Rs principles (reduce).
Experimental procedures	a. siRNA treatment: Mice were injected i.p.. with 0.2, 2 and/or 4 mg/kg of SiSaf-SiS sshLNP + *Clcn7*^{G215R}-siRNA or with SiS sshLNP Empty, 3 times a week for 2 or 4. For pilot experiments by i.p. administration mice were injected once with 4 mg/kg of SiSaf-SiS sshLNP + *Clcn7*^{G215R}-siRNA or with SiS sshLNP Empty and evaluated 48 hours thereafter. Treatments were done in the morning and ended before noon. Animals were monitored for further 3 hours.<br>b. Anesthesia: Ketamine/xylazine cocktail (87.5 mg/kg ketamine, 12.5 mg/kg xylazine) ophthalmic ointment was applied to both eyes to prevent desiccation. To recover from anesthesia mice were placed in warm, clean, dry, quiet environment away from other animals. Commercially available surgical heating pad were used to warm up animals. Bedding material was replaced with toweling material to avoid bedding to stick to eyes or be inhaled while animals were recovering from anesthesia.<br>c. Euthanasia: At the end of the experiments, mice were euthanized by CO_2 inhalation.
Experimental animals	Mice used for in vivo experiments were from the C57BL6/j strain, male, age of 10 days, weight 7± 3gr.<br>Genotype: WT and ADO2.<br>Immunocompetent.
Housing and husbandry	Animal facility: standard.<br>Temperature 20-24°C.<br>Diet: access to food and water *ad libitum*, normal diet (Mucedola code: 3KE25).<br>Dark/light cycle: 12/12 hours.<br>Humidity: 60 ± 5%<br>Cage: plastic.<br>Cage companions, 3 adults/cage, genders were not mixed.<br>Bedding material: high adsorbing power, without dust, changed every week.<br>Environmental enrichment was done with sterile material.
Sample size	Five-5 animals/group were used for the experiments.<br>The sample size was calculated for all the experiments using dedicated software (SigmaPlot v12), based on the expected differences.<br>The efficacy of the siRNA treatment allowed us to obtain significant results with relatively little sample sizes, according to the 3Rs principles.
Allocating animals to experimental groups	Animals were assigned to groups after randomization.

Experimental outcomes	To perform a systematic study to test the likelihood that the therapy could progress towards clinical trials.
Statistical	Statistical analysis was performed by the Student's *t*-test and Multiple Comparison one-way ANalysis Of VAriance (MC ANOVA) according to the type of data sets. The statistical methods are indicated in the figure and table legends. The p-values are indicated in the figures. A p value <0.05 was conventionally considered statistically significant.

Reference

1. Maurizi, A., Capulli, M., Patel, R., Curle, A., Rucci, N., Teti A. (2018). RNA interference therapy for autosomal dominant osteopetrosis type 2. Towards the preclinical development. Bone. *110*, 343-354. https//doi.org/10.1016/j.bone.2018.02.031.
