# Supplementary material for: Novel hybrid silicon-lipid nanoparticles deliver a siRNA to cure autosomal dominant osteopetrosis in mice. Implications for gene therapy in humans
Source: Mol Ther Nucleic Acids. 2023 Aug 19;33:925–37. doi: 10.1016/j.omtn.2023.08.020 (PMC10480457; doi:10.1016/j.omtn.2023.08.020)
Supplement: Document S2. Article plus supplemental information [file mmc2.pdf]

Molecular Therapy
Nucleic Acids
Original Article

Novel hybrid silicon-lipid nanoparticles deliver a siRNA to cure autosomal dominant osteopetrosis in mice. Implications for gene therapy in humans

Antonio Maurizi,[1] Piergiorgio Patrizii,[1,4] Anna Teti,[1] Flavia Maria Sutera,[2,4] Paulina Baran-Rachwalska,[2] Chris Burns,[3] Uttom Nandi,[2] Michael Welsh,[2] Nissim Torabi-Pour,[2] Ashkan Dehsorkhi,[2] and Suzanne Saffie-Siebert[2]

[1]Department of Biotechnological and Applied Clinical Sciences, University of L'Aquila, L'Aquila, Italy; [2]SiSaf Limited, Guildford, UK; [3]PorousSilicon, Salzburg, Austria

Rare skeletal diseases are still in need of proper clinically available transfection agents as the major challenge for first-in-human translation relates to intrinsic difficulty in targeting bone without exacerbating any inherent toxicity due to used vector. SiSaf's silicon stabilized hybrid lipid nanoparticles (sshLNPs) constitute next-generation non-viral vectors able to retain the integrity and stability of constructs and to accommodate considerable payloads of biologicals, without requiring cold-chain storage. sshLNP was complexed with a small interfering RNA (siRNA) specifically designed against the human *CLCN7*^{G215R} mRNA. When tested via single intraperitoneal injection in pre-puberal autosomal dominant osteopetrosis type 2 (ADO2) mice, carrying a heterozygous mutation of the *Clcn7* gene (*Clcn7*^{G213R}), sshLNP, this significantly downregulated the *Clcn7*^{G213R} related mRNA levels in femurs at 48 h. Confirmatory results were observed at 2 weeks and 4 weeks after treatments (3 intraperitoneal injections/week), with rescue of the bone phenotype and demonstrating safety. The pre-clinical results will enable advanced pre-clinical development of RNA-based therapy for orphan and genetic skeletal disorders by safely and effectively delivering biologicals of interest to cure human systemic conditions.

INTRODUCTION

Osteopetrosis, a rare bone genetic disorder characterized by high bone density, is known for encircling a group of altered skeletal phenotypes ascribable to an impairment of osteoclast activity. The autosomal dominant form of the disease (autosomal dominant osteopetrosis type 2 [ADO2]) is caused by single allele dominant negative mutations of the *CLCN7* gene, encoding the ClC-7 chloride transporter indispensable for the mechanism of bone resorption.[1] Although expert opinion-based guidelines for management of ADO2 have been published[2] and a promising small interfering RNA (siRNA)-based approach has been recently identified,[3,4] there is still an unmet need for developing suitable clinical therapies addressing bone gene-associated disorders, without resorting to bone marrow transplantation or the administration of off-label drugs.[2]

The treatment, as well as the prophylactic interventions, of genetic disorders and other pathologies such as cancer potentially require the introduction of nucleic acid specimens to a living organism to rebalance a clinical condition, immunize a subject, or ameliorate the effect of a chronic disease. Transfection of genetic material will usually occur through two main classes of vectors: viral and non-viral.

Currently, only six replicating-incompetent viral vectors have been authorized.[5,6] However, they still present several shortcomings, such as vaccine-induced immune thrombocytopenia and thrombosis.[6] Furthermore, subjects previously exposed to a viral vector can potentially raise an immune response against it, a phenomenon called "anti-vector immunity."[7]

Current viral strategies are based on adenoviruses (AVs), adeno-associated viruses (AAVs), and lentiviruses (LVs). Despite their successes in preclinical settings, many challenges still limit these approaches from attaining their full potential at the clinical level. Exogenous infections can occur in some instances with AV, while the majority of adverse events seem to be related to viral reactivation.[8] AAV limitations in clinical use are related to their immunogenicity or restricted gene packaging ability,[9,10] while LV show a high risk of insertional mutagenesis, mitigated but not fully eliminated by self-inactivating LV.[11]

Non-viral gene delivery systems are based on nanometric particles interacting with the negatively charged phosphate backbone of the nucleic acid and cationic lipids, peptides or other compounds,[12] which shield nucleic acid from degradation.[13] Various other cationic lipids combine a synthetic cationic lipid and a neutral lipid with cholesterol to generate a lipid nanoparticle (LNP) delivery system. They protect RNA up to certain degree from degradation.[14–16] However, LNPs are susceptible to degradation over time[17,18] and raise concerns for safety

Received 25 March 2023; accepted 16 August 2023;
https://doi.org/10.1016/j.omtn.2023.08.020.

[4]These author contributed equally

Correspondence: Anna Teti, Department of Biotechnological and Applied Clinical Sciences, University of L'Aquila, Via Vetoio - Coppito 2, 67100 L'Aquila, Italy.
E-mail: annamaria.teti@univaq.it
Correspondence: Suzanne Saffie-Siebert, SiSaf Limited, 3 Huxley Road, Surrey Research Park, Guildford, GU2 7RE, United Kingdom.
E-mail: saffie@sisaf.com

This is an open access article under the CC BY license (http://creativecommons.org/licenses/by/4.0/).

Table 1. Surface charge assessment at different ratios sshLNP-siRNA

Sample	sshLNP:siRNA	Size	PDI	ZP (mV)
sshLNP SiS + siRNA	20:1	194.8 ± 3.1	0.23 ± 0.03	+47.61 ± 1.13
sshLNP SiS + siRNA	12:1	142.7 ± 1.23	0.15 ± 0.01	+47.20 ± 0.35
sshLNP SiS + siRNA	8:1	144.0 ± 1.69	0.17 ± 0.02	+43.27 ± 0.75
sshLNP SiS + siRNA	6:1	143.3 ± 1.99	0.16 ± 0.02	−29.59 ± 1.06
sshLNP SiS + siRNA	4:1	143.7 ± 1.15	0.15 ± 0.01	−31.90 ± 0.79
sshLNP SiS + siRNA	2:1	147.2 ± 1.08	0.16 ± 0.02	−43.80 ± 0.74
Naked siRNA	–	–	–	−22.0 ± 0.30
Empty sshLNP	–	98.33 ± 0.54	0.08 ± 0.01	+55.43 ± 1.38
Si-NPs	–	–	–	−39.0 ± 0.41
Si-NPs doped	–	–	–	−27.5 ± 0.36

Zeta potential of siRNA, empty sshLNP, and Si-NPs are equally reported.

and stability, inducing acute immune responses, liver accumulation, or poor active targeting/selectivity.[19] Finally, polyethylenimines (PEIs) are highly cationic polymers that can result in toxicity, which precludes their use in clinical treatments.[7,18]

Although there has been a significant increase in demand for non-viral vector delivery systems in the last few years, several limitations persist. One of the most challenging is the safe targeting of skeletal tissue, for which no technology as such is clinically available to address bone diseases.

SiSaf Ltd has developed a biocompatible and biodegradable silicon-based drug delivery system for ocular gene therapy.[20] Using a similar approach, the company has developed a novel non-viral vector composed of mesoporous silicon nanoparticles functionalized with specific cationic moieties, neutral lipids, and a fraction of pegylated (PEG) lipids dispersed in an aqueous environment containing non-reducing disaccharide and amino acids for targeting bone tissue.[20] In this work, leading stabilized hybrid LNP (sshLNP) prototypes have been evaluated *in vivo* on an ADO2 mouse model harboring a *CLCN7*^{G215R} heterozygous mutation, representing a genetic bone disorder characterized by high bone mass and bone fragility.[21] We identified an approach by complexing the siRNA with sshLNP technology for an effective and safe long-term treatment with multiple dose regimens, thus being a key future prospect for skeletal gene therapy-based treatments. This mutation is caused by a G-A transition at human DNA position 21543 and at mouse DNA position 14365. It is the most frequent in-human ADO2 and leads to the G215R amino acid substitution in humans and G213R in mice.[21] Our siRNA recognizes and complements with similar efficacy and specificity both the human and mouse mutant mRNAs,[3] allowing us to test it in cells of both species and in ADO2 knock-in mice.[3,4]

RESULTS

sshLNP characterization

sshLNP characterization showed results in line with previous findings, reporting purity of more than 99% with other metal traces of less than 1% of total mass (evaluated by inductively coupled plasma-mass spectrometry [ICP-MS]), a spherical shape, and an average particle size of less than 100 nm (assessed by transmission electron microscopy [TEM]) and a mean surface area of approximately 34 m^2/g (determined by gas absorption and calculated through the Brunauer-Emmett-Teller [BET] theory).[20] The surface charge of sshLNP was determined to be −39.0 ± 1.3 mV and residual organic solvent analysis reported methanol levels below maximum International Council for Harmonization of Technical Requirements for Pharmaceuticals for Human Use limits (3,000 ppm) for both sshLNP and formulated silicon-based couriers.[20]

Characterization of the sshLNP delivery system

Seven sshLNP prototypes were formulated through surface functionalization of silicon nanoparticles with a variety of cationic moieties, additional helper and neutral lipids, and excipients commonly used for nucleic acid delivery intended for human applications, as shown in Table S1. SiS B sshLNP was formulated using the same ratios and composition of excipients as for SiS 2 sshLNP, with replacing non-doped silicon with doped silicon and by using siRNA with dAdT overhangs. All remaining prototypes were formulated with non-doped silicon and complexed with siRNA having dTdT overhangs.

Zeta potential and binding efficiency of sshLNP prototypes were tested at different ratios. Selected leading sshLNP prototypes reported surface charge, hydrodynamic size and PDI, as stated in Table 1. Zeta potential analysis on lead sshLNP prototypes revealed expected trend and inversion of surface charge based on ratio complexation siRNA:sshLNP (Table 1). For sshLNP siRNA complexes tested *in vivo*, the amount of accessible RNA determined via Ribogreen assay reported as 78.1% ± 1.7%, 44.3% ± 1.9%, and 5.1% ± 2.2% for 4 mg/kg, 2 mg/kg, and 0.2 mg/kg siRNA doses, respectively.

Stability of lead sshLNP prototypes over time showed that selected prototypes were able to retain surface charge over related counterpart formulated without silicon (Table 2).

Short-term *in vivo* treatments

For the *in vivo* experiments, it was first evaluated whether we could downregulate the *Clcn7*^{G213R}-mutant gene in our target organ, the bone, in a short-term experiment. To this end, 10-day-old ADO2 male mice were treated for 48 h with a single intraperitoneal (i.p.) injection of the selected sshLNP loaded with 4 mg/kg of *CLCN7*^{G215R}-siRNA. Each subject weighing 10 g received a dose containing 40 μg siRNA formulated within the sshLNP or ssLNP as such (empty sshLNP). At 48 h after injection, mice were sacrificed and RNA extracted from femurs was subjected to real time RT-PCR using primer pairs specific for the *Clcn7*^{G213R}, not recognizing the WT mRNA (Table S2).[3] Results showed that the sshLNP formulations, siRNA-SiS 2 and siRNA-SiS B, significantly downregulated the expression of the mutant *Clcn7*^{G213R} mRNA in the femurs of ADO2 mice (Figure 1A).

In addition, no adverse events or side effects were observed in all ADO2 mice treated with the sshLNP formulations. The treatment

Table 2. Zeta potential (ZP) values reported for carrier system formulated with lipids only and with sshLNP formulated with non-doped silicon and with doped silicon at room temperature

	0 weeks			2 weeks			4 weeks			12 weeks		
Sample	Size (nm)	PDI	ZP (mV)	Size (nm)	PDI	ZP (mV)	Size (nm)	PDI	ZP (mV)	Size (nm)	PDI	ZP (mV)
Lipids only	113.5	0.08	69	114.9	0.08	68	112.4	0.10	52	134.6	0.191	27.96
SIS non-doped silicon	94.28	0.234	52.71	103.9	0.230	53.19	96.5	0.200	54.15	87.14	0.201	53.19
SIS doped silicon	102.3	0.230	55.12	101.4	0.230	54.19	99.89	0.211	54.19	78.23	0.245	50.12

was well tolerated, and no topical reactions were observed at the injection site. After treatment, mice walking, feeding, and behavior were normal. No sign of suffering or weight loss was observed. At sacrifice, gross evaluation of visceral organs, including liver, kidney, spleen, and lung, did not reveal overt alterations of the organ size and macroscopic morphology. No mice died during the treatment. Overall, all the SiS sshLNP formulations tested in this short-term protocol were safe without overt side effects evidently impacting the health of the treated ADO2 mice.

To further evaluate the effect of adding a positive charge to silicon through a doping process on retaining transfection efficiency, SiS 2 and SiS B sshLNP were selected as leading candidates, renamed SiS12 and SiS13 sshLNP, respectively, and further tested on a 2-week dose regimen, with 3 i.p. injections per week after being both complexed with *CLCN7*^{G215R} siRNA having dTdT overhang. The results obtained from the gene expression analysis showed that both siRNA-SiS12 and siRNA-SiS13 candidates significantly downregulated the expression of the mutant *Clcn7*^{G213R} mRNA in the femurs (Figures 1B and 1C) of ADO2 mice, compared with the empty SiS12, while a prominent effect on downregulating the expression of the mutant gene in peripheral blood mononuclear cells (PBMCs) was highlighted for siRNA-SiS12 (Figure 1D) over the siRNA-SiS13 (Figure 1E). The evaluation of the serum bone resorption marker, collagen type 1 C-telopeptide (CTX), unveiled a trend of increase in mice treated with both siRNA-SiS12 and siRNA-SiS13 vs. empty SiS12 and SiS13, respectively (Figures 1F and 1G).

Additional statistical comparisons between siRNA-SiS12 and siRNA-SiS13 formulations were carried out to better discriminate the efficacy of the two sshLNP. Results showed that both were equivalent on the CTX serum level and the *Clcn7*^{G213R} mRNA downregulation in the femurs (Table S3). Of note, siRNA-SiS12 formulation was more effective in downregulating the mutant *Clcn7*^{G213R} mRNA in PBMCs compared with SiS13-siRNA (Table S3).

No adverse events or side effects were observed in mice treated with the siRNA-SiS12 and siRNA-SiS13 formulations. The multiple dose administration regimen was well tolerated, and no change of behavioral stereotypes or variation in food intake were observed. The gross evaluation of visceral organs (liver, kidneys, spleen, brain, heart, and lungs) did not reveal alterations of size, weight (Figures 2A and 2B), or macroscopic morphology when compared with untreated wild-type (WT) mouse organs. No deaths were reported during treatment.

Although both sshLNPs were shown to be effective in downregulating the expression of mutated gene in target tissue, based on these results and intending to obtain a circulating biomarker to be potentially used for *in vivo* monitoring of treatment progression for this particular study, the siRNA-SiS12 formulation was selected to be further investigated.

In vitro stability of SiS12 sshLNP and cellular uptake

Before performing the complete *in vivo* tests, we evaluated the stability and the uptake efficiency of SiS12 sshLNP in a series of *in vitro* studies. First, to track stability by imaging, we incubated the AD293 cells for 24 h with our test nanoparticles loaded with the siGlO green, fluorescent indicator.[22] As a reference transfection reagent, we used lipofectamine (LPF). Over a treatment time of 24 h with freshly prepared complexes (Figure S1; Table S4, time 0 siGLO-nanoparticle incubation), we observed that naked siGLO was not internalized by cells, while the siGLO+LPF complex showed very low transfection efficiency. siGLO+LNPs were instead internalized by cells with medium efficiency, while siGLO+fresh LPF was transfected with higher efficiency. Importantly, siGLO+SiS12 sshLNP showed the highest internalization potential compared with the other conditions (Figure S1A).

When siGLO complexes were incubated for 24 h at 4°C (Figure S1B), room temperature (22°C) (Figure S1C), or 37°C (Figure S1D) before being administered to cells, siGLO+SiS12 sshLNP retained stability in all conditions, followed by naked siGLO+fresh LPF and siGLO+LNP (Table S4). In contrast, siGLO+LPF complex remained inefficient.

The stability of the formulation at 4°C, 22°C and 37°C was confirmed in the mouse osteoclast precursor cell line, RAW264.7 cells, incubated with 100 nM *CLCN7*^{G215R} siRNA complexed with SiS12 labeled with the a green fluorescent tag, containing 18:1 of 1,2-dioleoyl-sn-glycero-3-phosphoethanolamine-N-carboxyfluorescein (PE-CF) at 10% of the total amount of DOPE used for formulating the Bio-Courier (SiS12-18:PE-CF10%) (Figure 3A). Furthermore, the uptake in this cell line was dose dependent (Figure 3B) and increased with time, reaching a plateau after 24 h of incubation (Figure 3C), further confirming stability. Taken together, these results underline the potential of this formulation to represent a stable product suitable for safe storage.

Bone and bone marrow cell uptake

To address which cell types in the bone take up the siRNA-S12S formulation, we flushed out bone marrow cells from femurs and

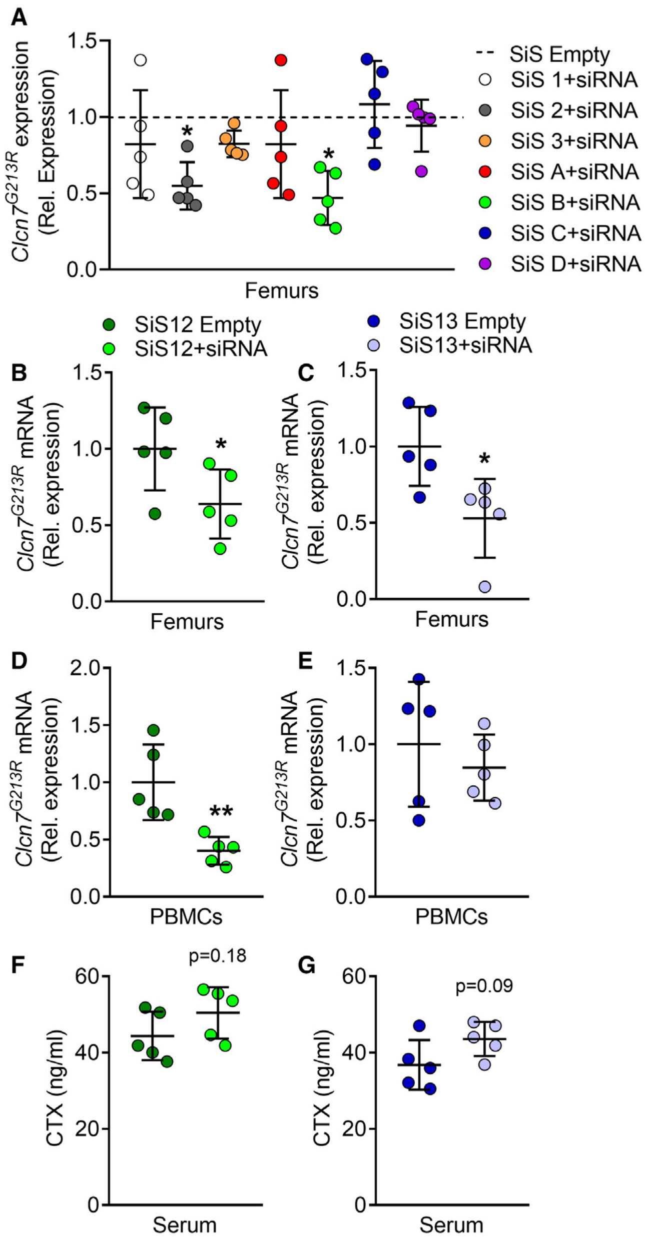

Figure 1. $Clcn7^{G213R}$ gene expression and serum CTX analysis in ADO2 mice treated with SiS sshLNP formulations

Ten-day-old ADO2 male mice were treated i.p. with the indicated SiS sshLNP loaded with 4 mg/kg $CLCN7^{G215R}$-siRNA or empty. (A) Mice were treated for 48 h and real time RT-PCR was performed using a specific primer pair for the mutant $Clcn7^{G213R}$ (Table S2), normalized by *Gapdh*. (B and C) Mice were treated for 2 weeks and real time RT-PCR for the mutant $Clcn7^{G213R}$ was performed in femurs and (D and E) in PBMCs normalized by *Gapdh*. (E and F) Evaluation of serum CTX in ADO2 mice treated for 2 weeks. Data represent the mean ± SD of n = 5 ADO2 mice per group. Student's t test vs. the SiS empty sshLNP group. $^{*}p \leq 0.05$; $^{**}p < 0.01$.

incubated them for 3 h with 100 nM siRNA formulated with SiS12 sshLNP conjugated with the green fluorescent tag 18:1PE-CF10%. Cells were allowed to adhere to culture dish for 12 min to separate adherent cells from non-adherent cells. Then we evaluated the green fluorescence by fluorometry on 500,000 cells per group, observing that non-adherent cells, known to be enriched in the hematopoietic cell component, which includes the osteoclast lineage, showed twice as much fluorescence compared with adherent cells, the latter of which is known to be enriched in the stromal cell component, which includes the osteogenic lineage (Figure 3D). One million labeled non-adherent cells were then sorted by fluorescence-activated cell sorting for size and granularity to distinguish the populations enriched in lymphocytes, monocytes, and granulocytes (Figures 3E and 3F). Among them, the highest fluorescence was observed in the monocyte-enriched fraction (Figure 3G), known to include the osteoclast precursors, representing our *in vivo* target bone cells to treat osteopetrosis.

Biodistribution and pharmacokinetics

To evaluate the biodistribution and pharmacokinetics of the siRNA-SiS12 formulation, we used 4 mg/kg $CLCN7^{G215R}$ siRNA in the complex with the SiS12 tagged with 18:1PE-CF10%. Four-week-old WT mice were injected i.p. once with the formulation and sacrificed after 6, 24, and 48 h. After sacrifice, organs were collected and freshly analyzed to detect the green fluorescence of the SiS12 sshLNP. Results showed a modest fluorescence in serum at 6 h, with a subsequent decline, suggesting a rapid transit in the circulation. In contrast, the fluorescence increased over time in the other organs, reaching the maximum at 24 h followed by a plateau (Figure 3H). Femurs, representing our target bone segments in this experiment, showed a mean fluorescence uptake at 48 h of 13% of the administered dose. Fluorescence was also observed in liver, kidneys, lungs, and spleen (Figure 3H; Table S5).

To evaluate the impact of the route of administration on biodistribution and pharmacokinetics, we also treated the mice by subcutaneous injection with the same protocol as for the i.p. administration, observing similar results, with femur uptake at 48 h of approximately 19% of the administered dose (Figure 3I; Table S5).

Long-term *in vivo* experiments

Having demonstrated the efficacy and tolerability of the siRNA-SiS12 formulation in short-term *in vivo* experiments, and efficacy and stability in *in vitro* tests, we performed *in vivo* long-term experiments to evaluate the effect of siRNA-SiS12 on the bone phenotype of 10-day-old ADO2 male mice treated i.p. 3 times a week for 4 consecutive weeks. WT mice treated with saline were used as control to compare the phenotype of the ADO2 mice with normal reference values. Mice treated with the $CLCN7^{G215R}$-siRNA naked or combined with the commercially available PEI derivative, in-vivoJetPEI[3,4] were used as negative and positive controls, respectively. The treatment was performed according to Table S6. At the end of the experiment, mice were sacrificed, and bones, organs, and blood were harvested for analysis.

Bone morphometry and quality

Micro computed tomography (μCT) analysis carried out on proximal tibias revealed that ADO2 mice treated with SiS12 sshLNP combined with 4 and 2 mg/kg of $CLCN7^{G215R}$-siRNA showed a rescue of the

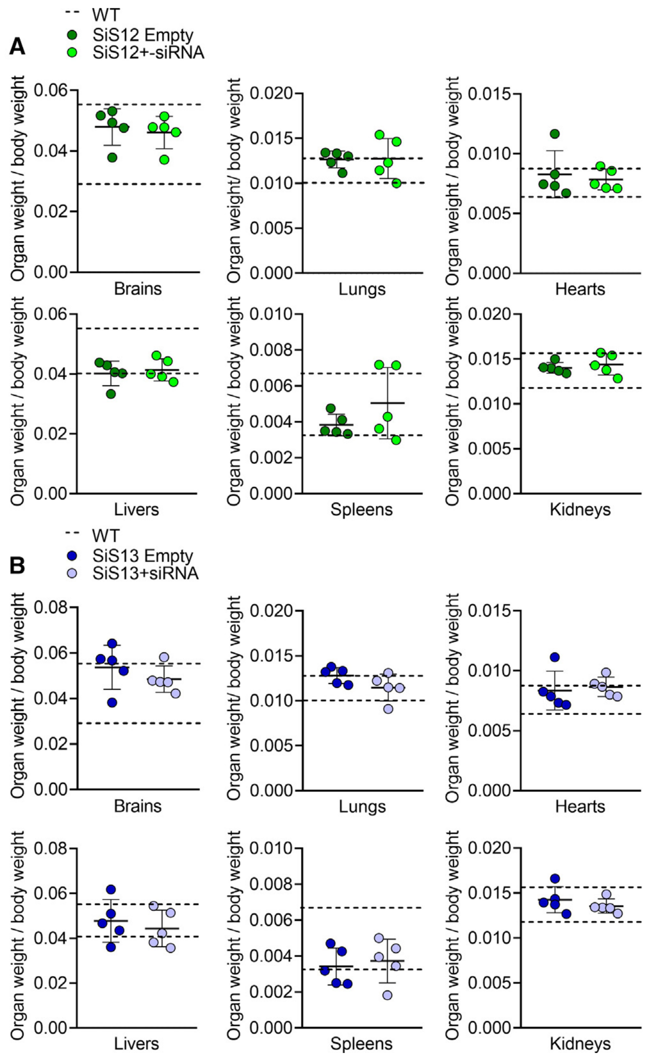

Figure 2. Treatment with SiS12 and Sis13 formulations for 2 weeks

Ten-day-old ADO2 male mice were treated i.p. 3 times a week for 2 weeks with SiS12 or SiS13 sshLNP complexed with 4 mg/kg of *CLCN7*^{G215R}-siRNA or as such (empty sshLNP). (A and B) At the end of the experiment, the weights of the indicated organs were taken to assess the presence of macroscopic alterations. For each mouse, the organs' weight was normalized for the body weight. An untreated WT group was examined for comparison. Data are the mean ± SD of n = 5 mice per group. Student's t test.

bone (Figure 4A), with trabecular bone structural variables returning to the levels of WT mice. In fact, the analysis showed a significant decrease of trabecular bone volume over total tissue volume (Figure 4B), associated with a decrease of trabecular number (Figure 4C) and an increase of trabecular separation (Figure 4D), in the mice treated with 4 and 2 mg/kg of *CLCN7*^{G215R}-siRNA combined with SiS12 sshLNP compared with empty SiS12 sshLNP. Of note, all mentioned variables returned to the WT level. Trabecular thickness was unremarkable (Figure 4E), in agreement with the observation that this variable is not affected by the disease.[21] The level of efficacy of 4 mg/kg siRNA-SiS12 formulation was comparable with that of the positive control formulation at the same dose of siRNA. No effect on trabecular bone structural variables was observed with 0.2 mg/kg *CLCN7*^{G215R}-siRNA combined with SiS12 sshLNP or with 4 mg/kg naked *CLCN7*^{G215R}-siRNA (Figures 4A–4E) compared with ADO2 mice treated with empty SiS12.

At the same time, bone quality analysis was carried out using the micro indentation apparatus Biodent. The indentation test on the midshaft of the femurs harvested from the treated mice was performed for calculating the indentation distance and the total indentation distance. These parameters are inversely correlated with bone strength and quality. The results revealed that the indentation distance (Figure 4F) and the total indentation idstance (Figure 4G) were significantly lower in the ADO2 mice treated with the SiS12 sshLNP combined with 4 and 2 mg/kg of *CLCN7*^{G215R}-siRNA compared with the SiS12 empty sshLNP, indicating an increase in bone strength that was rescued to the WT level. Of note, the effect of 4 mg/kg siRNA-SiS12 formulation was comparable with that of the positive control at the same dose of siRNA. No effect on bone quality was observed with 0.2 mg/kg *CLCN7*^{G215R}-siRNA combined with the SiS12 sshLNP, or with 4 mg/kg naked *CLCN7*^{G215R}-siRNA, compared with ADO2 mice treated with SiS12 sshLNP empty for the considered route of administration (Figures 4F and 4G).

Clcn7^{G213R} gene downregulation in bone and PBMCs

RNA was isolated from femurs and PBMCs of ADO2-treated mice and the downregulation of the mutant *Clcn7*^{G213R} mRNA was evaluated by real time RT-PCR using primers specific for the mutant *Clcn7*^{G213R} (Table S2).[3] The results in femurs showed a significant downregulation of the mutant *Clcn7*^{G213R} in ADO2 mice treated with 4 mg/kg *CLCN7*^{G215R}-siRNA combined with the SiS12 sshLNP or with the positive control compared with ADO2 mice treated with empty SiS12 sshLNP (Figure 4H). Moreover, a trend of reduction was also observed in ADO2 mice treated with 2 and 0.2 mg/kg *CLCN7*^{G215R}-siRNA combined with the SiS12 sshLNP, while the administration of naked *CLCN7*^{G215R}-siRNA significantly increased the *Clcn7*^{G213R} gene expression in the femurs of ADO2, compared with empty SiS12 sshLNP (Figure 4H).

In the PBMCs isolated from ADO2 mice treated with SiS12 sshLNP combined with 4, 2, and 0.2 mg/kg of *CLCN7*^{G215R}-siRNA we observed a significant downregulation of the mutant *Clcn7*^{G213R} compared with the empty SiS12 sshLNP (Figure 4I). Similar *Clcn7*^{G213R} downregulation was also observed in the PBMCs of ADO2 mice treated with 4 mg/kg *CLCN7*^{G215R}-siRNA combined with the positive control (Figure 4I).

Safety analysis

No obvious adverse events or side effects were observed in all ADO2 mice treated with the naked *CLCN7*^{G215R}-siRNA and siRNA-SiS12 formulations. The treatment was well tolerated, and no topical reactions were observed at the injection site. After treatments, mice locomotion, food intake, and behavior were normal. No sign of suffering

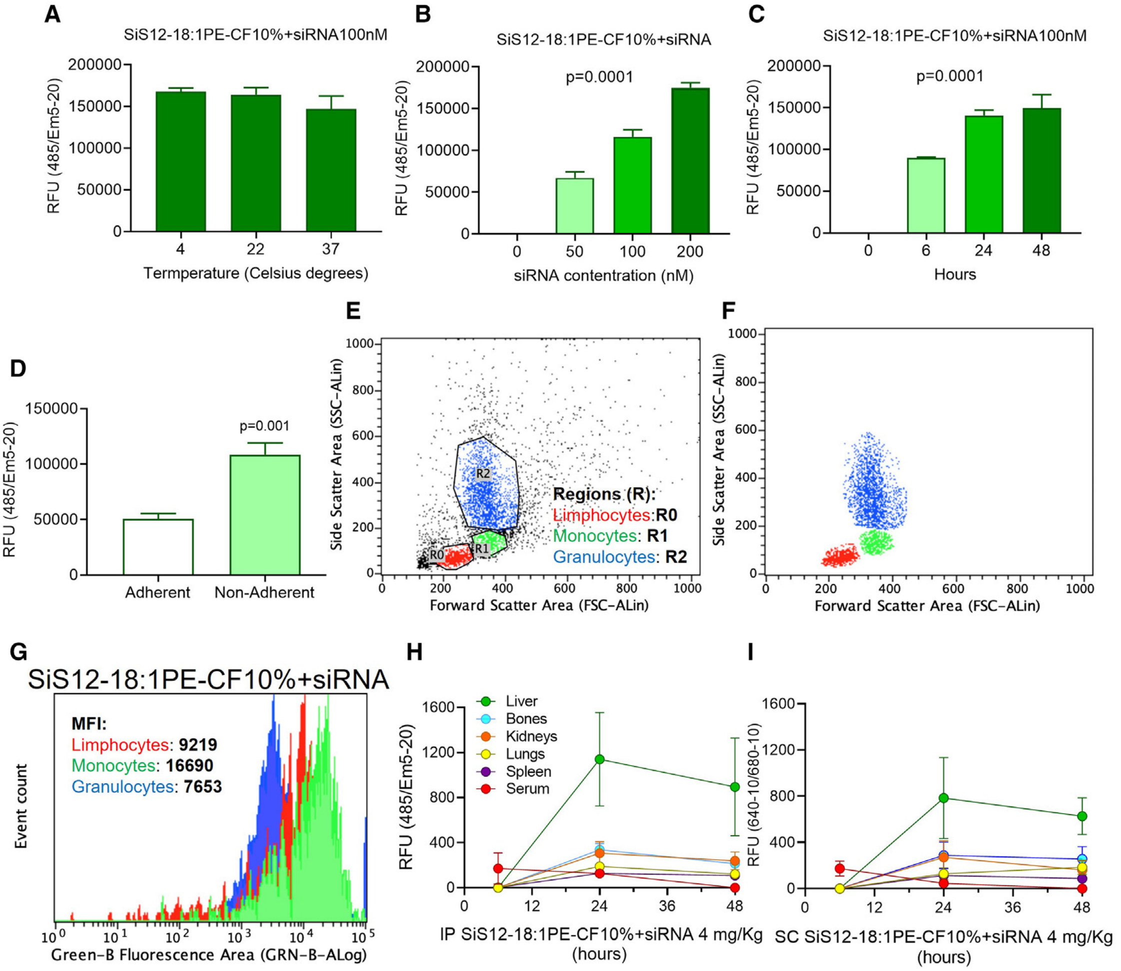

Figure 3. Cellular uptake, biodistribution, and pharmacokinetics

RAW264.7 cells were incubated with SiS12-18:1PE-CF10%+siRNA at the indicated (A) temperatures (time: 24 h), (B) concentrations (time: 24 h) and (C) times and evaluated for cellular uptake by fluorometry. (D) Cells were flushed out from femurs and allowed to adhere to culture dish for 12 min to separate the adherent fraction (enriched in stromal cells) from the non-adherent fraction (enriched in hematopoietic cells). Five hundred thousand cells per fractions were then incubated with SiS12-18:1PE-CF10%+siRNA for 3 h, washed, and evaluated by fluorometry to detect the green fluorescence. (E) One million of labeled non-adherent cells were sorted by fluorescence-activated cell sorting (FACS) by size and granularity to distinguish the fractions enriched in lymphocytes, monocytes and granulocytes. (F) Image in (E) cleaned from the background to better visualize the three fractions. (G) Mean fluorescence intensity (MFI) in the cell fractions shown in (G). (H) Four-week-old ADO2 mice were injected i.p. and (I) subcutaneously with 4 mg/kg ADO2 siRNA formulated with SiS12-18:PE-CF10%). Mice were sacrificed at the indicated time points, and organs were harvested and analyzed by fluorometry to detect green fluorescence. Results are the mean ± SD of 3 *in vitro* experiments or 5 mice per group. Statistics: (B and C) multiple comparison ANOVA; (F) Student's t test.

or weight loss was observed in ADO2 mice treated with the naked siRNA and the siRNA-SiS12 formulations compared with WT mice treated with saline (Figure 5A). At sacrifice, body weight and size, and weight of treated ADO2 visceral organs, including liver, kidneys, spleen, brain, heart, and lungs, were not altered compared with WT mice (Figures 5B–5H). No mice died during the treatment (Figures 5B–5H). Of note, ADO2 mice treated with 4 mg/kg of *CLCN7*^{G215R}-siRNA combined with the positive control showed a lower body weight gain during the treatment and, at sacrifice, the body weight was significantly lower compared with empty SiS12 sshLNP-treated ADO2 mice and WT mice (Figures 5A and 5B). In contrast, brain, lung, and kidney weights, normalized by body weights, were higher than control (Figures 5C, 5D, and 5H), suggesting a possible toxic effect of the positive control confirming previous observations.[23] Finally, serological disease biomarkers of liver (alanine aminotransferase and alkaline phosphatase) (Figure 5I) and kidney (urea) (Figure 5K) were unchanged in all treated ADO2 mice compared with the WT controls.

The siRNA-SiS12 formulations did not significantly alter the transcriptional expression in femurs of the inflammatory cytokines *Tnf-α*, *Il-1β*, *Il-6*, and *Ifn-γ* (Figures 6A–6D) and of the closely related chloride transporters, *Clcn3* and *Clcn5* (Figures 6E and 6F), nor were significant differences observed in the serum levels of the

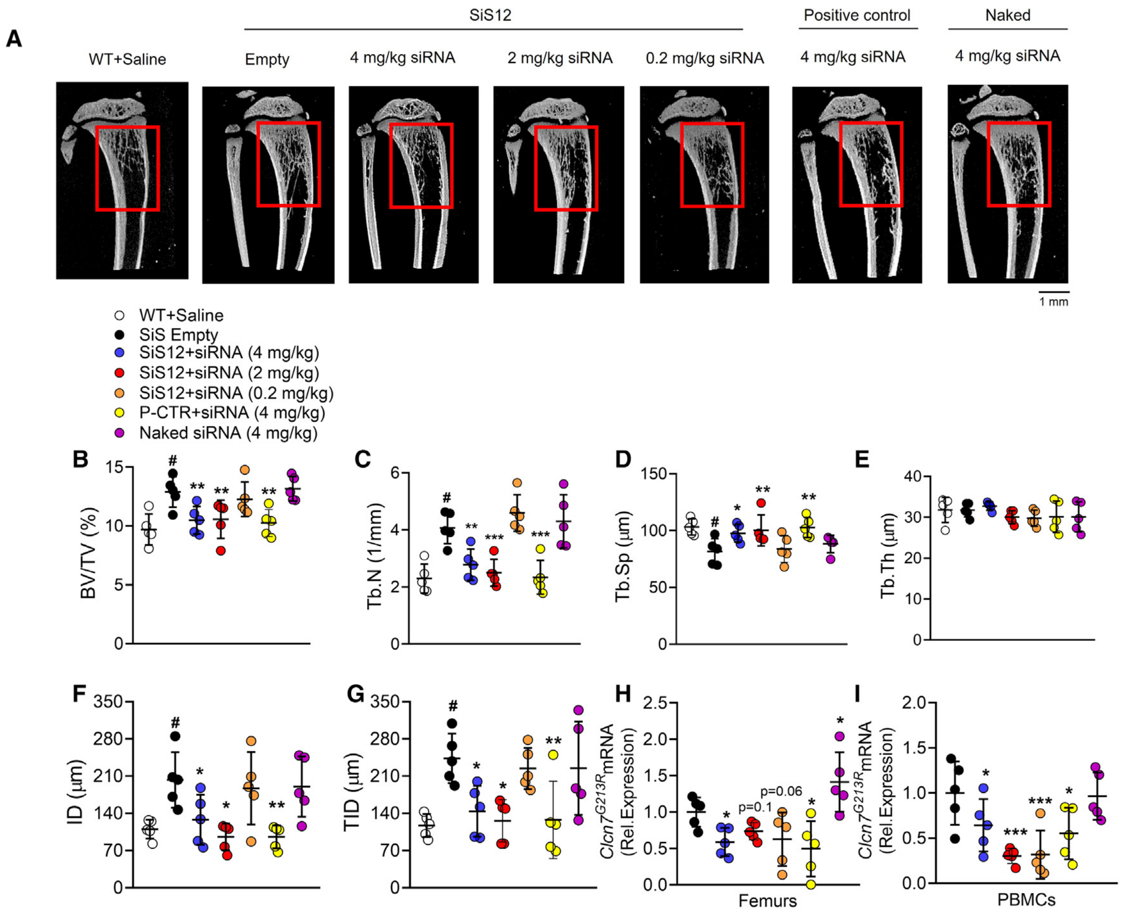

Figure 4. Bone phenotype analysis

Ten-day-old ADO2 male mice were treated with the indicated siRNA and siRNA-SiS sshLNP or positive control (P-CTR) complexes and doses, 3 times a week for 4 weeks. At the end of the experiment, mice were sacrificed, and tibias and femurs were collected and analyzed. (A) Representative μCT reconstructions of proximal tibias. Red squares indicate the region of interest subjected to the analysis. (B) Morphometric analysis of bone volume over total volume (BV/TV%) and (C) trabecular number (Tb.N), (D) separation (Tb.Sp), and (E) thickness (Tb.Th) assessed by μCT. (F) Indentation distance (ID) and (G) total indentation distance (TID) measured in femur midshafts by indentation test using the Biodent device. (H) Downregulation of the mutant *Clcn7*^{G213R} in femurs and (I) PBMCs, normalized by *Gapdh*. Results are (A) representative or (B–I) the mean ± SD of n = 5 mice per group. Multiple comparison ANOVA. *$p < 0.05$; **$p < 0.01$; ***$p < 0.001$ vs. the SiS empty sshLNP group; $^{\#}p < 0.01$ vs. WT + Saline group.

inflammatory cytokines tumor necrosis factor (TNF)-α and IL-6 (Figures 6F and 6G) and of the antibodies against PEG liposome products vs. WT-saline mice (Figure 6H).

DISCUSSION

In this study, a siRNA therapy has been optimized to treat an autosomal dominant bone disease (ADO2) characterized by the impaired activity of the chloride/proton antiporter type 7, essential for charge balancing the environment of the resorption lacuna excavated by osteoclasts during the process of bone resorption.[1] Autosomal dominant diseases are ideal candidates for siRNA therapy, provided that the curative siRNAs are properly designed to complement the mutant site of the affected heterozygous mRNA without affecting the WT mRNA. Indeed, this is the case for our therapy, for which we have identified a highly efficient and specific siRNA, complementing the mRNA encoded by the ADO2 mutant *CLCN7* gene.

In this study, we provided evidence that a formulation of our ADO2 siRNA complexed with sshLNPs proved safe and effective in downregulating the expression of the mutant *Clcn7*^{G213R} mRNA in ADO2 mice, rescuing the ADO2 bone mass to the WT level. Importantly, the formulation also improved bone quality, which is degraded in ADO2 patients, causing numerous atraumatic fractures that are difficult to heal.[24] The relevance of these observations is several folds, since these results open an avenue to the exploitation of this discovery for a first-in-human clinical approach.

From a technological perspective, several considerations emerged from our study. Chemically modified siRNAs are currently approved for therapy.[25] Of note, we did not increase siRNA stability by chemical modifications because they are reported to be associated with toxicity, immune stimulation, and off-target effects.[26] Moreover, an increased stability of the siRNA is known to increase resistance to

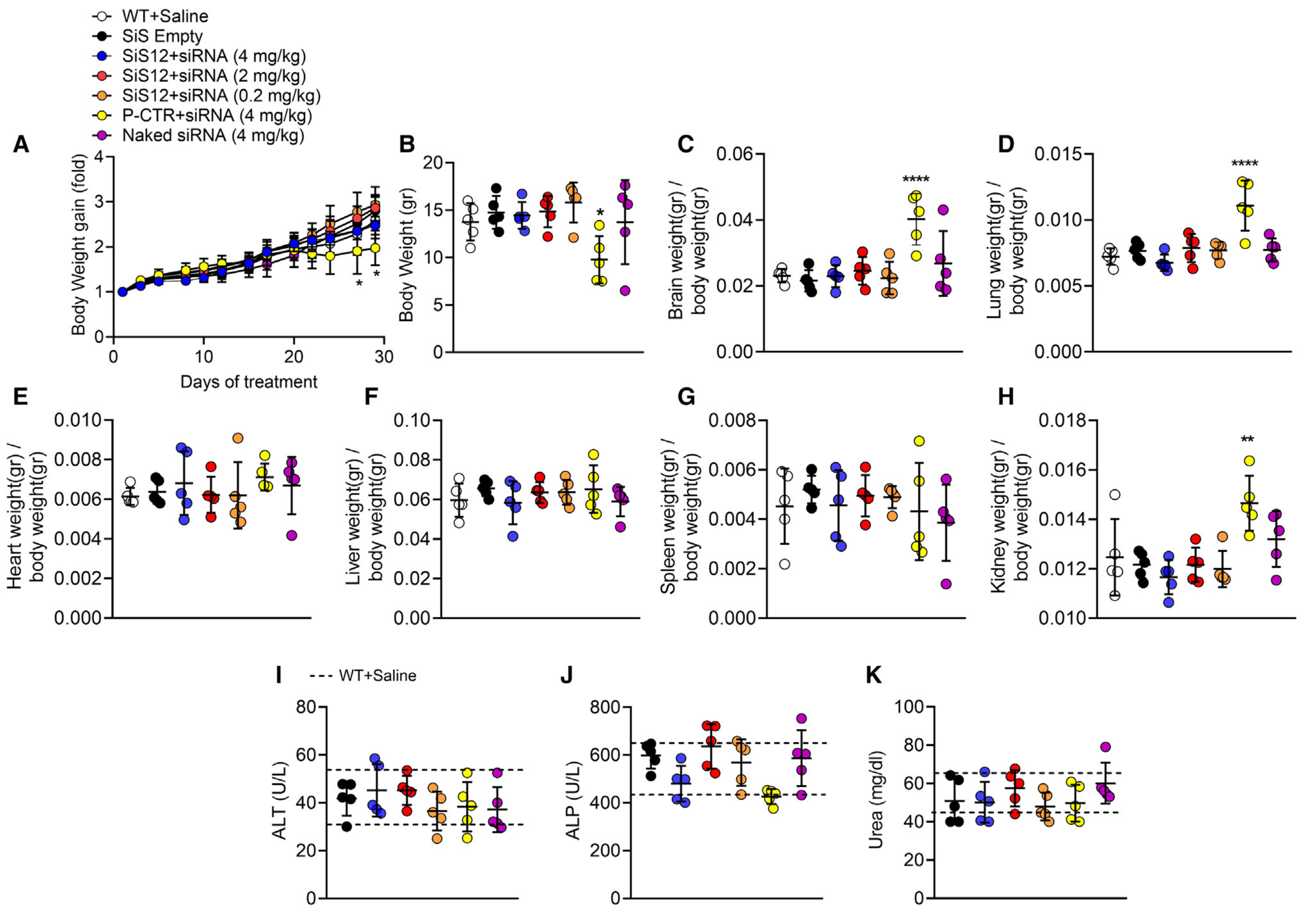

Figure 5. Safety analysis

Ten-day-old ADO2 male mice were treated with the indicated siRNA and siRNA-SiS sshLNP or positive control (P-CTR) complexes and doses, 3 times a week for 4 weeks. (A) Body weights measured at the indicated time points and their gain calculated normalizing the body weights measured during the treatment with the body weights at time = 0 (pre-treatment). (B) Body weights and weights of (C) brains, (D) lungs, (E) hearts, (F) livers, (G) spleens, and (H) kidneys assessed at the end of the experiment. (I and J) Sera collected from the same mice were analyzed by the Reflotron method for the indicated biomarkers of liver and (K) kidney diseases. Results are the mean ± SD of n = 5 mice per group. Multiple comparison ANOVA vs. the WT + Saline group. *p < 0.05; **p < 0.01; ****p < 0.0001.

cleavage, which induces altered response to the RISC complex in both short- and long-term dose regimens. To avoid these adverse events, we developed the SiS sshLNP technology that protects the efficacy of siRNA without need for chemical modifications.

Using the sshLNP technology, we performed *in vivo* screening of many *CLCN7*^{G215R}siRNA-SiS sshLNP complexes from a formulative perspective. Comparing SiS 2 and SiS 3 sshLNP, although cationic lipid DOTAP is present and the total amount of lipids is unvaried between the two, the change of helper moieties affected the transfection efficiency, leading to the absence of statistical significance between treated and control mice for the SiS 3 group. Furthermore, the presence of PEI groups, integrated to helper lipids only (SiS C) or in the presence of DOTAP (SiS D), did not sustain the transfection in the presence of siRNA dTdT overhangs for the intended sequence. This outcome resembles the lack of efficacy of other non-viral vectors constituted by polycationic PEI groups, complexed with siRNA possessing alternative overhangs with respect to sticky 3′ overhangs dAdT.[27] Substitution of positive charge (SiS A, SiS C vs. SiS 2) or alteration of helper lipid composition (SIS 3 vs. SIS 2) had a similar impact on the efficiency of transfection. In comparison, the identified leading candidates, SiS 2 and SiS B, formulated with dTdT overhangs or dAdT overhangs, retained transfection efficiency.

Our short-term (48 h) and medium-term (2 weeks) *in vivo* experiments using the SiS 2 (renamed SiS12) and SiS B (renamed SiS13) sshLNP allowed us to refine the selection of the formulations, taking the SiS12 as lead sshLNP prototype to be investigated in long-term (4 weeks) experiments. To this end, we first tested the stability *in vitro* of the siRNA-SiS12 formulation, noting no significant impairment upon 24 h incubation of the complex at 4°C, room temperature (22°C), and 37°C. The uptake was dose and time dependent and remained stable for 48 h both *in vitro* and *in vivo*. Furthermore, the

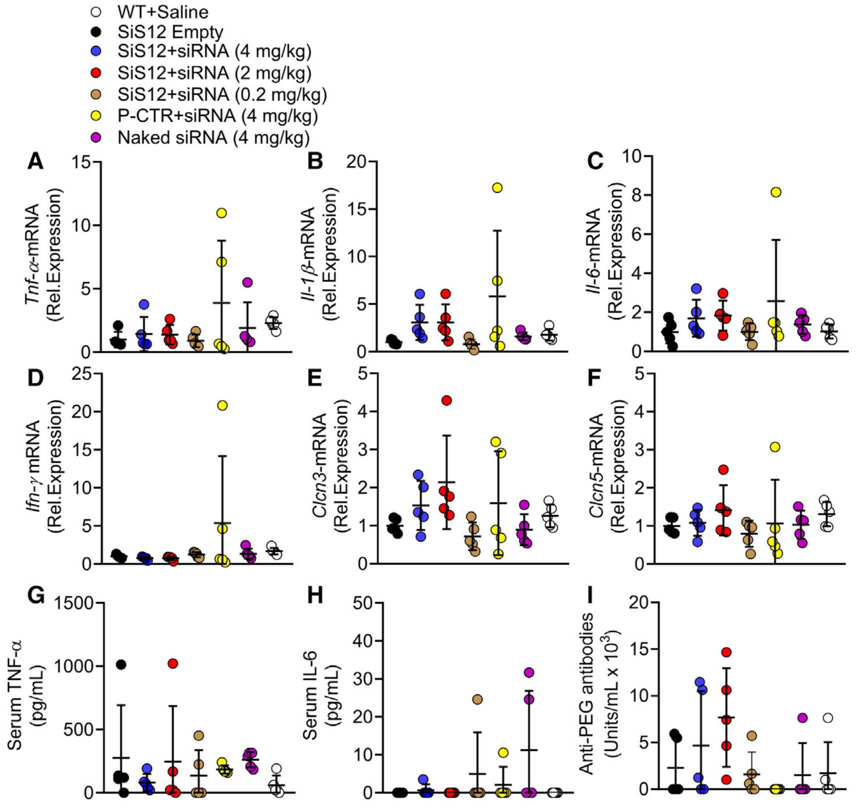

Figure 6. Expression of inflammatory cytokines, chloride transporters and anti-PEG antibodies

Ten-day-old ADO2 male mice were treated with the indicated siRNA and siRNA-SiS sshLNP or positive control (P-CTR) complexes and doses, 3 times a week for 4 weeks. (A) Real-time RT-PCR of the inflammatory cytokines *Tnf-α*, (B) *IL-1β*, (C), *IL-6*, and (D) *INF-γ* expressed in femurs, normalized by *Gapdh*. (E) Real time RT-PCR of the chloride transporters *Clcn3* and (F) *Clcn5* expressed in femurs, normalized by *Gapdh*. (G) Serum levels of TNF-α, (H) IL-6 and (I) anti-PEG antibodies. Results are the mean ± SD of n = 5 mice per group. MC-ANOVA vs. the WT+saline group. Differences are statistically not significant.

formulation was biodistributed *in vivo* systemically in bones and other organs, representing a strength for a potential use in humans, given that the *CLCN7* gene is expressed in many organs, proved to alter organ function when mutated, and to be susceptible in ADO2 to down-regulation and phenotypic rescue when targeted by our siRNA.[28]

Then, we administered 0.2 to 4 mg/kg siRNA-SiS12 complex in ADO2 mice for a complete phenotypic evaluation in comparison to the commercially available positive control, with which the efficacy of the *CLCN7*^{G215R}-siRNA was assessed in our previous studies.[3,4] This treatment proved to be effective, with a dose-dependent improvement in bone structural variables and bone quality. The maximal effect was observed already at 2 mg/kg siRNA dose, which is one-half of the effective dose of the reference positive control formulation.[3] The *CLCN7*^{G215R}-siRNA-SiS12 complex downregulated the mutant *Clcn7*^{G213R} gene in femurs and PBMCs, including circulating mononuclear osteoclast precursors.

Importantly, in contrast with the *CLCN7*^{G215R}-siRNA-positive control complex, no toxic effects were observed injecting the *CLCN7*^{G215R}-siRNA-SiS12 complex. No topical reactions were noticed, while walking ability, food intake, and body weight gain were unchanged compared with WT mice treated with saline and with ADO2 mice treated with naked siRNA or SiS12 empty sshLNP. Similar unchanged weights were observed in all single visceral organs investigated in treated mice vs. controls. In contrast, the weight of brain, lung, and kidney, normalized to body weight, was higher in mice treated with the *CLCN7*^{G215R}-siRNA-positive control complex. Furthermore, the *CLCN7*^{G215R}-siRNA-SiS12 complex did not exert pro-inflammatory effect, as demonstrated by the unchanged expression of *Tnf-α*, *IL-1β*, *IL-6*, and *INF-γ* and their serum proteins when detectable. No changes were also observed in the mRNA expression of the *Clcn7* closely related chloride transporters, *Clcn3* and *Clcn5*, suggesting high specificity for the *Clcn7* mRNA and lack of off-target's events. Finally, anti-PEG antibodies were detectable in the serum of our control WT mice, and their levels were not statistically different vs. the treated groups. This result is consistent with,[29,30] who detected anti-PEG antibodies in healthy control mice without specific exposure to PEG.

Altogether, these results suggest that the *CLCN7*^{G215R}-siRNA -SiS12 complex is effective, safe, and stable in our murine model of ADO2, suggesting potential for first in human translation to the ADO2 patients.

In conclusion, genetic skeletal disorders are still challenging to be treated because of the lack of tissue specificity reported for most drug delivery systems with respect to bone targeting.[31] We here demonstrated that a siRNA-based approach targeting the *CLCN7*^{G215R} mRNA, when vehiculated through SiSaf's sshLNPs, is able to significantly downregulate the *Clcn7*^{G213R} expression in femurs. The complexed sshLNP leading prototype induced a significant downregulation of the mutant mRNA in PBMCs of treated ADO2 subjects, thus providing an additional translational circulating marker for ADO2 treatment toward clinical development. Further analyses revealed no adverse alterations in treated mice, demonstrating excellent tolerability and achievement of intended therapeutic outcome. Reported results have significant translational impact on orphan and rare bone disease therapies and open the path to human trials using sshLNP technology with RNA.

MATERIALS AND METHODS

Preparation and characterization of silicon nanoparticles

Silicon nanoparticles (Si-NPs) were sourced as silicon nanopowder, purity ≥98% (American Elements, non-doped material; PorousSilicon, doped material). These were assessed in preliminary studies and determined to be suitable for use, using procedures and techniques previously described.[14–16,32] Si-NPs were preliminarily subjected to methanol rinsing and then a slow evaporation technique, until dryness reached. Afterward, the Si-NPs were analyzed for determining silicon content, available surface area, particle size, and residual solvent before further formulation process. ICP-MS analysis was performed to determine the concentration of 28Si in the Si-NPs as described in.[20]

The surface area was determined by subjecting particles to gas sorption using Quantachrome Nova 2200e and values were calculated applying the BET theory. Before the measurements, the samples were degassed at 350°C overnight. To visually examine structure and size of Si-NPs, particles were suspended at approximately 0.01% in deionized water and a drop of sample was dried at reduced temperature under vacuum over a carbon-coated copper grid for analysis via TEM. Accelerating voltage of 100 kV under FEI Talos L120C G2 TEM was then applied for observing samples.

Preparation of sshLNP formulations

The sshLNP formulations were prepared with various compositions, using rotary evaporation technique followed by extrusion process. All chemicals used in the preparation were purchased from Sigma-Aldrich unless stated otherwise.

Formulations were prepared starting with a lipid thin film generated by means of solvent evaporation and hydrated with Si-NPs aqueous suspension. Specifically, required amounts of lipids were transferred into a round bottom flask and the solvent was carefully evaporated using rotary evaporator with a water bath set at 40°C under vacuum. The thin lipid film was then hydrated using hydrating suspension containing the required amounts of silicon and water dispersible excipients in a water bath set at 60°C (amounts stated in the results section). The sshLNP were rested in the fridge (4°C) overnight and were then extruded through polycarbonate membrane filters of the pore sizes 0.4 μm and 0.1 μm (10 times each) at 60°C (Avanti Polar Lipid Extruder, equipped with Whatman polycarbonate filters). Prepared samples were stored at standard refrigerated conditions until usage.

SiS12 sshLNP was used in formulations for *in vivo* biodistribution/pharmacokinetics experiments and for *in vitro* tests on RAW264.7 cells were labeled with 10% w/w of 18:1 PE-CF (Avanti Polar Lipids).

Residual solvent analysis for sshLNP

sshLNP formulations were analyzed for residual solvents content as previously described.[20] For headspace gas chromatography, 1 mL of the sample was weighed accurately into a headspace vial (weight of the 1 mL is recorded and critical for concentration calculations) and the sample vial was capped with a magnetic lid. The analysis was performed using Shimadzu GC-2030 connected to a flame ionization detector (FID) and an AOC-6000 autosampler, with a Phenomenex Zebron ZB-624plus 30 m × 0.32 mm × 1.80 μm column. Autosampler and headspace conditions were: incubation 80°C for 20 min, syringe 90°C, 250 rpm, pre-purge time 30 s, and injection flow rate 10 mL/min. Injector conditions were: hydrogen as the gas carrier, 140°C, split ratio 5:1, and carrier gas linear speed 35 cm/s. Oven temperature was: baseline at 40°C for 12 min, then ramp/rate 16.7 °C/min for 12 min, and then hold at 240°C for 12 min. FID temperature was 250°C, nitrogen flow 30 mL/min, and air flow 400 mL/min. For blank measurement, deionized water was used, and standard curves for methanol (75–4,500 ppm) quantification were prepared from reference material purchased from Sigma Aldrich (United States Pharmacopeia [USP] Residual Solvent Class 2 – Methanol Reference Standard), properly diluted with deionized water for reaching desired concentrations. The limit of methanol in any sample must be below 3,000 ppm to fulfill USP method 467 criteria.

Silicon assessment for sshLNP

sshLNP were analyzed for silicon content post filtration by means of Shimadzu ICPE-9820 plasma atomic emission spectrometer. Briefly, empty sshLNP samples were vortexed to ensure homogeneity, and an aliquot of 10 mL was transferred to a 15-mL polypropylene tube and sonicated for 1.5 h. All samples were prepared and analyzed in triplicate, with imposing a software input of 1 for dilution rate. Concentration of silicon in samples was measured against a calibration curve generated by diluting the Periodic Table Mix 1 for ICP certified standard (Sigma Aldrich, UK) with type 1 ultrapure water, with standards prepared as per dilutions. Parameters setting for ICPE-9820 were as follows: carrier gas 0.7 L/min, auxiliary gas 0.6 L/min, plasma gas 10 L/min, radiofrequency power 1.2 kW, exposure time 30 s, view direction axial, element/Wv = Si/251.61 nm, solvent/sample rinse time 30–60 s, and peristaltic pump ranging from 20 to 60 rpm.

Complexation of siRNA to sshLNP

In this study, two different siRNAs were used: standard ADO2 siRNA with dTdT overhang and ADO2 siRNA with dAdT overhang, which served as a positive control (Table 1).

The selected siRNA was complexed with commercial preclinical transfection agent, in-vivoJetPEI (JetPEI), as previously described.[3,4] Both siRNAs were designed as 21-mers with a central 19-bp duplex region and above specified dinucleotide overhangs on each 3′ end (Table S7). These were chemically synthesized and annealed by Horizon Discovery. For nucleic acid complexation with sshLNP formulations, siRNA dissolved in nuclease-free water was added to the aqueous suspension of nanoparticles and incubated at room temperature for 60 min before further analysis. To examine the complexation ratio, siRNA was mixed with sshLNP at different w/w ratios and analyzed by gel electrophoresis.

Gel retardation assay

To assess the formation of complexes between the siRNA and sshLNP, nanoparticles suspension in nuclease-free water was

combined with siRNA at various loading ratios (i.e., 1:2, 1:4, 1:6, 1:8, 1:10, 1:12, or 1:20 w/w of siRNA to sshLNP) and analyzed by electrophoresis on a 1% agarose gel (E-Gel agarose gels [1%];Invitrogen, ThermoFisher Scientific) for 7 min at 100 V. Equal amount of 150 ng siRNA was loaded into each well. Gels were run and visualized using E-Gel Power Snap Electrophoresis Device and camera (Invitrogen, ThermoFisher Scientific). As marker, E-Gel 1Kb plus Express DNA ladder (Invitrogen, ThermoFisher Scientific) was used.

Dynamic light scattering measurement

Mean hydrodynamic particle size, polydispersity index (PDI) and surface charge (zeta-potential) of siRNA complexes and empty sshLNP were determined using ZetaSizer Nano ZS (Malvern P Analytical). For measuring the particle size and PDI, 20 μL of each sample was mixed with 980 μL nuclease free water and placed inside a single use DLS cuvette (DT0012, Malvern). Each sample was measured four times and the average of the last three runs is reported. For measuring the zeta potential, 150 μL of each sample was diluted either with 850 μL nuclease-free water to give a total of 1,000 μL and filled inside a folded capillary cell (DTS1070, Malvern) using a 1-mL graded syringe, avoiding introducing any bubbles in the capillary, to reach yield count rate in a range of 100–400 kcps. Measurements were performed at 25°C in triplicate and reported as mean ± SD. Instrumental settings were: absorption = 0.001, refractive index = 1.33, dispersant = water, viscosity = 0.8872 at 25°C, and time of equilibration 120 = sec.

Accessible siRNA assay

To evaluate the amount of accessible siRNA within the complexes, the Quant-iT RiboGreen RNA reagent from Invitrogen (ThermoFisher) was used, according to the manufacturer's guideline. For analysis, Varioskan LUX multimode plate reader (ThermoFisher) was used at an excitation wavelength of 480 nm and an emission wavelength of 520 nm. The test was performed in 1× Tris-EDTA (TE)-buffer (10 mM Tris-HCl and 1 mM EDTA).

Cell culture

The AD293 human embryonic kidney cells (Life Technologies) were cultured in DMEM (ThermoFisher) enriched with 10% fetal bovine serum (ThermoFisher). All cell cultures were incubated at 37°C in the presence of 5% CO_2 and passaged following standard laboratory protocols.

Cell transfection efficiency assessed by fluorescence measurement

For this assessment, sshLNP were complexed to a fluorescent commercially available model siRNA, siGLO Green Transfection Indicator (Dharmacon, Horizon). AD293 cells were seeded at 7×10^3 cells per well in a 96-well plate and grown overnight in a cell incubator at 37°C with 5% CO_2. Transfection experiments were performed 24 h later. The siGLO samples (kept at different storage conditions after preparation, specifically 4°C, room temperature and 37°C over 0 and 24 h) were diluted in OptiMEM reduced-serum cell culture medium (ThermoFisher) and added to the cells in n = 6 replicates at final 100 nM siGLO concentration (10 pmol–133 ng per 100 μL growth medium per well). Medium only (untransfected control for background fluorescence) and 10 pmol siGLO complexed with 0.2 μL LPF 2000 transfection reagent (Invitrogen) as a positive control, were prepared alongside at same 100 nM final concentration. After 24 h of incubation at standard culture conditions, the cells were washed with PBS (Sigma Aldrich) and stained with Hoechst 33342 fluorescent nuclear dye (NucBlue Live reagent, ThermoFisher Scientific) diluted in FluoroBrite DMEM medium (ThermoFisher). The fluorescence intensity of siGLO was measured at Ex/Em wavelength 485/520nm using well-scanning mode on Varioskan LUX multimode plate reader (ThermoFisher). To standardize siGLO FI data, the number of cells per well was assessed by nuclear fluorescence measurement at Ex/Em wavelength 360/460 nm, and the value of siGLO/Hoechst fluorescence intensity was calculated.

Imaging of transfected cells by fluorescence microscopy

AD293 cells were seeded at 5×10^4 cells per well on a 24-well plate with coverslips and grown in a cell incubator at 37°C with 5% CO_2 overnight. Transfection experiments were performed 24 h later. The siGLO samples were diluted in OptiMEM reduced-serum cell culture medium (ThermoFisher) and added to the cells in duplicates at final 100 nM siGLO concentration (50 pmol–665 ng per 500 μL growth medium per well). As a positive control, 50 pmol siGLO was complexed with 1 μL LPF and used for transfection at same 100 nM final siGLO concentration. After 24 h of incubation at standard culture conditions, the cells were washed with PBS (Sigma Aldrich), fixed with 4% paraformaldehyde (ThermoFisher) in PBS, and counterstained with DAPI in UltraCruz Aqueous Mounting Medium (Santa Cruz Biotechnology). Imaging was performed using Zeiss Model AxioScope A.1 LED Fluorescent Illumination Microscope equipped with DAPI (365 nm) and GFP (470/40 nm) filter and Zeiss AxioCam MRc digital camera (Carl Zeiss).

Animals

The *Clcn7*$^{G213R/WT}$ ADO2 mouse model (*Mus Musculus*, C57/BL6 background) has been described by Alam et al., 2013,[21] and was previously used by Capulli et al.[3] and Maurizi et al.[4] All *in vivo* experiments were conducted in compliance with applicable national and international guidelines and policies (European Economic Community Council Directive 86/609, OJ L 358, 1, December 12, 1987; Italian Legislative Decree 4.03.2014, n.26, *Gazzetta Ufficiale della Repubblica Italiana*) and were approved by the Italian Ministry of Health (approval n. 112/2020-PR). The study was performed according to the Animal Research Reporting of In Vivo Experiments (ARRIVE, Table S8) guidelines, with n = 5 mice/group upon randomization. Mice were injected i.p. with 50–100 μL of the solution containing the siRNA-sshLNP complex at doses and frequencies described in the text and in the figure legends. At the end of treatments, mice were humanely sacrificed by CO_2 inhalation after anesthesia.

Isolation of PBMCs and serological tests

Blood sampling was performed in the morning in fasted mice, according to Christgau.[33] Buffy-coat mononuclear cells were isolated from

diluted blood (1:1 in Hanks' solution) layered over Histopaque 1077 (Sigma Aldrich) solution and centrifuged at 400×*g* for 30 min.

Sera were used to measure levels of the bone resorption marker CTX by ELISA kit (IDS), following the manufacturers' instructions. Serum biomarkers of liver (alanine aminotransferase and alkaline phosphatase) and kidney (urea) diseases were measured using Reflotron strips (Roche).

Real time RT-PCR

Total RNA was extracted from tissues using Trizol (ThermoFisher) according to the manufacturer's instructions. RNA was quantified by NanoDrop (ThermoFisher) and quality checked by 1% agarose gel run. One microgram of RNA was reverse transcribed into cDNA using the RevertAid H Minus First Strand cDNA Synthesis Kit (ThermoFisher). Real-time PCR reaction was performed loading 0.1 μg of cDNA using the Luna Universal qPCR Master Mix (New England Biolabs). Gene expression data were represented as relative expression over control, normalized by *Gapdh*. Primer sequences and PCR conditions are listed in Table S2.

μCT analysis

Tibias were fixed in 4% paraformaldehyde for 48 h and scanned, by μCT SkyScan 1174, with a 6.- μm resolution using an X-ray voltage of 50 kV. The Skyscan Nrecon software was used to reconstruct the images using a modified Feldkamp algorithm.[34] Three-dimensional analysis was carried out with a Marching Cubes type model with a rendered surface.[35] The trabecular bone parameters were calculated on 550 consecutive slides starting from 100 μm below the growth plate. Pratt's algorithm was used to take two-dimensional measurements. Threshold values were applied for segmenting trabecular bone, and structural variables were determined according to Bouxsein et al.[36]

Indentation tests

Mechanical tests were performed on the midshaft of the femurs using the Reference Point Indentation technique by Biodent. Bones were kept in ice-cold PBS during the tests to maintain tissue hydration. Tests were performed using 5–10 indentation cycles at 2 Hz with a force of 2 N. The indentation distance and total indentation distance were calculated for each test.

Statistical analyses

Statistical analyses were performed in line with the recommendations of experimental design and analysis in Pharmacology.[37] All results are presented as mean ± SD of n = 3 experiments per cell preparations or n = 5 mice/group. Statistical analyses were carried out by the Prism by GraphPad v7.0, software, using Student's t test or multiple comparisons one-way ANOVA. The statistical methods are indicated in the figure and table legends, and the p values threshold was 0.05.

DATA AND CODE AVAILABILITY

The authors confirm that the data supporting the findings of this study are available within the article and its supplemental materials. *In vivo* data are available from University of L'Aquila with the permission of SiSaf Ltd. Restrictions apply to the availability of these data, which were developed under License for this study.

SUPPLEMENTAL INFORMATION

Supplemental information can be found online at https://doi.org/10.1016/j.omtn.2023.08.020.

ACKNOWLEDGMENTS

This study has been supported by a License Agreement between SiSaf Ltd and the University of L'Aquila. A.M. is supported by the "Piano Operativo Nazionale (PON) Ricerca Innovazione" (FSE-REACT EU D.M.1062).

AUTHOR CONTRIBUTIONS

A.M. contributed to the conceptual and experimental design of the *in vivo* experiments, the evaluation of the results and the manuscript writing. P.P. performed the *in vivo* experiments, evaluated the results, prepared the graphs and the study reports. A.T. contributed to the coordination of the activities, the conceptual and experimental design, the evaluation of the results, and the manuscript writing. F.M.S., N.T.-P., P.B.R., M.W., A.D., and S.S.S. contributed to the conceptual and experimental design of the SiS sshLNP. F.M.S., N.T.-P., P.B.R, and A.D. performed in silico and *in vitro* evaluation of SiS sshLNP. F.M.S., C.B., U.N., A.D., and S.S.S. contributed to the manufacturing setting of sshLNP's production. F.M.S., P.B.R., A.D., and S.S.S. Evaluated results, elaborated data, and contributed to manuscript writing. All the authors reviewed the text and agreed with the final manuscript.

DECLARATION OF INTERESTS

A.T. and A.M. served as consultant, P.P had a service agreement, and F.M.S., N.T.-P., P.B.R, C.B., U.N., M.W., and A.D. are scientists working for SiSaf Ltd, a commercial stage biopharmaceutical company. S.S.-S. is founder and CEO of SiSaf Ltd.

REFERENCES

1. Cleiren, E., Bénichou, O., Van Hul, E., Gram, J., Bollerslev, J., Singer, F.R., Beaverson, K., Aledo, A., Whyte, M.P., Yoneyama, T., et al. (2001). Albers-Schönberg disease (autosomal dominant osteopetrosis, type II) results from mutations in the ClCN7 chloride channel gene. Hum. Mol. Genet. *10*, 2861–2867. https://doi.org/10.1093/hmg/10.25.2861.

2. Wu, C.C., Econs, M.J., Di Meglio, L.A., Insogna, K.L., Levine, M.A., Orchard, P.J., Miller, W.P., Petryk, A., Rush, E.T., Shoback, D.M., et al. (2017). Diagnosis and management of osteopetrosis: consensus guidelines from the osteopetrosis working group. J. Clin. Endocrinol. Metab. *102*, 3111–3123. https://doi.org/10.1210/jc.2017-01127.

3. Capulli, M., Maurizi, A., Ventura, L., Rucci, N., and Teti, A. (2015). Effective small interfering RNA therapy to treat CLCN7-dependent autosomal dominant osteopetrosis type 2. Mol. Ther. Nucleic Acids *4*, e248. https://doi.org/10.1038/mtna.2015.21.

4. Maurizi, A., Capulli, M., Patel, R., Curle, A., Rucci, N., and Teti, A. (2018). RNA interference therapy for autosomal dominant osteopetrosis type 2. Towards the preclinical development. Bone *110*, 343–354. https://doi.org/10.1016/j.bone.2018.02.031.

5. Zhao, Z., Anselmo, A.C., and Mitragotri, S. (2022). Viral vector-based gene therapies in the clinic. Bioeng. Transl. Med. *7*, e10258. https://doi.org/10.1002/btm2.10258.

6. Hviid, A., Hansen, J.V., Thiesson, E.M., and Wohlfahrt, J. (2022). Association of AZD1222 and BNT162b2 COVID-19 vaccination with thromboembolic and thrombocytopenic events in frontline personnel: A retrospective cohort study. Ann. Intern. Med. *175*, 541–546. https://doi.org/10.7326/M21-2452.

7. Lopez-Gordo, E., Podgorski, I.I., Downes, N., and Alemany, R. (2014). Circumventing antivector immunity: potential use of nonhuman adenoviral vectors. Hum. Gene Ther. *25*, 285–300. https://doi.org/10.1089/hum.2013.228.

8. Lion, T. (2019). Adenovirus persistence, reactivation, and clinical management. FEBS Lett. *593*, 3571–3582. https://doi.org/10.1002/1873-3468.13576.

9. Bulcha, J.T., Wang, Y., Ma, H., Tai, P.W.L., and Gao, G. (2021). Viral vector platforms within the gene therapy landscape. Signal Transduct. Targeted Ther. *6*, 53. https://doi.org/10.1038/s41392-021-00487-6.

10. Au, H.K.E., Isalan, M., and Mielcarek, M. (2021). Gene therapy advances: A meta-analysis of AAV usage in clinical settings. Front. Med. *8*, 809118. https://doi.org/10.3389/fmed.2021.809118.

11. Milone, M.C., and O'Doherty, U. (2018). Clinical use of lentiviral vectors. Leukemia *32*, 1529–1541. https://doi.org/10.1038/s41375-018-0106-0.

12. Butt, M.H., Zaman, M., Ahmad, A., Khan, R., Mallhi, T.H., Hasan, M.M., Khan, Y.H., Hafeez, S., Massoud, E.E.S., Rahman, M.H., and Cavalu, S. (2022). Appraisal for the potential of viral and nonviral vectors in gene therapy: A review. Genes *13*, 1370. https://doi.org/10.3390/genes13081370.

13. Mellott, A.J., Forrest, M.L., and Detamore, M.S. (2013). Physical non-viral gene delivery methods for tissue engineering. Ann. Biomed. Eng. *41*, 446–468. https://doi.org/10.1007/s10439-012-0678-1.

14. Durymanov, M., and Reineke, J. (2018). Non-viral delivery of nucleic acids: insight into mechanisms of overcoming intracellular barriers. Front. Pharmacol. *9*, 971. https://doi.org/10.3389/fphar.2018.00971.

15. Aston, R., Saffie-Siebert, R., Canham, L., and Ogden, J. (2005). Nanotechnology applications for drug delivery. Pharmaceut. Technol. Eur. *17*, 21–28.

16. Saffie-Siebert, R., Ogden, J., and Parry-Billings, M. (2005). Nanotechnology approaches to solving the problems of poorly water-soluble drugs. Drug Discov. World *6*, 71–76.

17. Siepmann, J., Faham, A., Clas, S.D., Boyd, B.J., Jannin, V., Bernkop-Schnürch, A., Zhao, H., Lecommandoux, S., Evans, J.C., Allen, C., et al. (2019). Lipids and polymers in pharmaceutical technology: Lifelong companions. Int. J. Pharm. *558*, 128–142. https://doi.org/10.1016/j.ijpharm.2018.12.080.

18. D'Amico, C., Fontana, F., Cheng, R., and Santos, H.A. (2021). Development of vaccine formulations: past, present, and future. Drug Deliv. Transl. Res. *11*, 353–372. https://doi.org/10.1007/s13346-021-00924-7.

19. Han, X., Zhang, H., Butowska, K., Swingle, K.L., Alameh, M.G., Weissman, D., and Mitchell, M.J. (2021). An ionizable lipid toolbox for RNA delivery. Nat. Commun. *12*, 7233. https://doi.org/10.1038/s41467-021-27493-0.

20. Baran-Rachwalska, P., Torabi-Pour, N., Sutera, F.M., Ahmed, M., Thomas, K., Nesbit, M.A., Welsh, M., Moore, C.B.T., and Saffie-Siebert, S.R. (2020). Topical siRNA delivery to the cornea and anterior eye by hybrid silicon-lipid nanoparticles. J. Contr. Release *326*, 192–202. https://doi.org/10.1016/j.jconrel.2020.07.004.

21. Alam, I., Gray, A.,K.,, Chu, K., Ichikawa, S., Mohammad, K.S., Capannolo, M., Capulli, M., Maurizi, A., Muraca, M., Teti, A., et al. (2014). Generation of the first autosomal dominant osteopetrosis type II (ADO2) disease models. Bone *59*, 66–75. https://doi.org/10.1016/j.bone.2013.10.021.

22. Hurtado, C., Safarova, A., Smith, M., Chung, R., Bruyneel, A.A.N., Gomez-Galeno, J., Oswald, F., Larson, C.J., Cashman, J.R., Ruiz-Lozano, P., et al. (2019). Disruption of NOTCH signaling by a small molecule inhibitor of the transcription factor RBPJ. Sci. Rep. *9*, 10811. https://doi.org/10.1038/s41598-019-46948-5.

23. Breunig, M., Lungwitz, U., Liebl, R., and Goepferich, A. (2007). Breaking up the correlation between efficacy and toxicity for nonviral gene delivery. Proc. Natl. Acad. Sci. USA *104*, 14454–14459. https://doi.org/10.1073/pnas.0703882104.

24. Waguespack, S.G., Hui, S.L., Dimeglio, L.A., and Econs, M.J. (2007). Autosomal dominant osteopetrosis: clinical severity and natural history of 94 subjects with a chloride channel 7 gene mutation. J. Clin. Endocrinol. Metab. *92*, 771–778. https://doi.org/10.1210/jc.2006-1986.

25. Hu, B., Zhong, L., Weng, Y., Peng, L., Huang, Y., Zhao, Y., and Liang, X.J. (2020). Therapeutic siRNA: state of the art. Signal Transduct. Targeted Ther. *5*, 101. https://doi.org/10.1038/s41392-020-0207-x.

26. Selvam, C., Mutisya, D., Prakash, S., Ranganna, K., and Thilagavathi, R. (2017). Therapeutic potential of chemically modified siRNA: Recent trends. Chem. Biol. Drug Des. *90*, 665–678. https://doi.org/10.1111/cbdd.12993.

27. Bolcato-Bellemin, A.L., Bonnet, M.E., Creusat, G., Erbacher, P., and Behr, J.P. (2007). Sticky overhangs enhance siRNA-mediated gene silencing. Proc. Natl. Acad. Sci. USA *104*, 16050–16055. https://doi.org/10.1073/pnas.0707831104.

28. Maurizi, A., Capulli, M., Curle, A., Patel, R., Ucci, A., Côrtes, J.A., Oxford, H., Lamandé, S.R., Bateman, J.F., Rucci, N., and Teti, A. (2019). Extra-skeletal manifestations in mice affected by Clcn7-dependent autosomal dominant osteopetrosis type 2 clinical and therapeutic implications. Bone Res. *7*, 17. https://doi.org/10.1038/s41413-019-0055-x.

29. Kozma, G.T., Shimizu, T., Ishida, T., and Szebeni, J. (2020). Anti-PEG antibodies: Properties, formation, testing and role in adverse immune reactions to PEGylated nano-biopharmaceuticals. Adv. Drug Deliv. Rev. *154–155*, 163–175. https://doi.org/10.1016/j.addr.2020.07.024.

30. Chen, B.M., Cheng, T.L., and Roffler, S.R. (2021). Polyethylene glycol immunogenicity: Theoretical, clinical, and practical aspects of anti-polyethylene glycol antibodies. ACS Nano *15*, 14022–14048. https://doi.org/10.1021/acsnano.1c05922.

31. Malcolm, D.W., Wang, Y., Overby, C., Newman, M., and Benoit, D.S.W. (2020). Delivery of RNAi-based therapeutics for bone regeneration. Curr. Osteoporos. Rep. *18*, 312–324. https://doi.org/10.1007/s11914-020-00587-2.

32. Saffie-Siebert, S.2015-R. (2015). Delivery System Comprising a Silicon-Containing Material, U.S. Pat. No. 9,132,083. https://patents.google.com/patent/US9132083B2/en.

33. Christgau, S. (2000). Circadian variation in serum CrossLaps concentration is reduced in fasting individuals. Clin. Chem. *46*, 431.

34. Feldkamp, L.A., Davis, L.C., and Kress, J.W. (1984). Practical cone-beam algorithm. J. Opt. Soc. Am. *1*, 612–619.

35. Lorensen, W.E., and Cline, H.E. (1987). Marching cubes: a high resolution 3d surface construction algorithm. SIGGRAPH Comput. Graph. *21*, 163–169.

36. Bouxsein, M.L., Boyd, S.K., Christiansen, B.A., Guldberg, R.E., Jepsen, K.J., and Müller, R. (2010). Guidelines for assessment of bone microstructure in rodents using micro-computed tomography. J. Bone Miner. Res. *25*, 1468–1486. https://doi.org/10.1002/jbmr.141.

37. Festing, M.F.W., and Altman, D.G. (2002). Guidelines for the design and statistical analysis of experiments using laboratory animals. ILAR J. *43*, 244–258. https://doi.org/10.1093/ilar.43.4.244.

OMTN, Volume *33*

Supplemental information

Novel hybrid silicon-lipid nanoparticles deliver a siRNA to cure autosomal dominant osteopetrosis in mice. Implications for gene therapy in humans

Antonio Maurizi, Piergiorgio Patrizii, Anna Teti, Flavia Maria Sutera, Paulina Baran-Rachwalska, Chris Burns, Uttom Nandi, Michael Welsh, Nissim Torabi-Pour, Ashkan Dehsorkhi, and Suzanne Saffie-Siebert

Supplemental Information

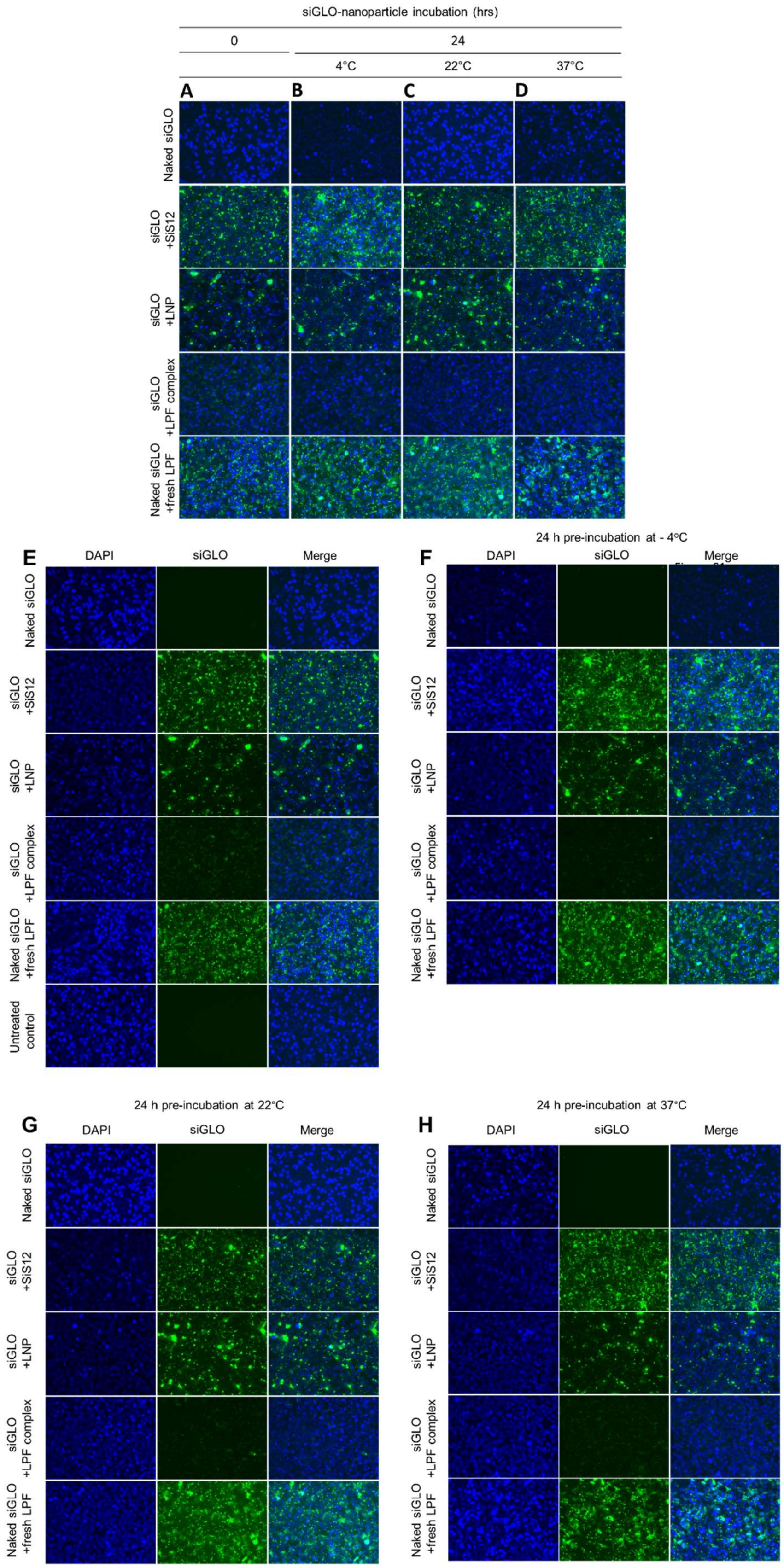

Figure S1. Assessment of cellular uptake of siGLO. Green-fluorescent siGLO (50 pmol) was complexed with SiS12 sshLNP prototype, lipid nanoparticles (LPN) or Lipofectamine (LPF) reagent and administered to AD293 cells **(A)** freshly prepared (time 0) or after incubation for 24 hrs at **(B)** 4°C, **(C)** 22°C or **(D)** 37°C. Treated AD293 cells were then cultured for 24 hrs at 37°C, in a humified atmosphere of 5% CO_2. Naked siGLO was subjected to the same treatment as control. For positive transfection control, a freshly prepared Lipofectamine (LPF) complex with siGLO (siGLO+fresh LPF) was used. At the end of incubation, cells were washed, fixed with 4% paraformaldehyde, stained with DAPI nuclear dye (blue) and imaged by fluoresce microscopy under 200x magnification. Pictures are representative of 3 experiments with n=6 replicates per condition and show merged images. **(E-H)** Separate blue and green images alongside merge images.

Table S1. Composition of all sshLNP prototypes tested.

Prototypes	Lipids				Silicon	Glycine	Trehalose	PoLyLysine	PEI (5)
	DOTAP (1)	DOPE (2)	mPEG2000-DSPE (3)	DPPC (4)					
**SiS 1**	7.25	7.30	1.45	NA(6)	2	1	2	NA	NA
**SiS 2**	7.25	7.30	1.45	NA	1	0.5	1	NA	NA
**SiS 3**	7.45	3.55	1.45	3.55	1	0.5	1	NA	NA
**SiS A**	NA	11.25	4.75	NA	1	0.5	1	3.2	NA
**SiS B**	7.25	7.30	1.45	NA	1	0.5	1	NA	NA
**SiS C**	NA	11.25	4.75	NA	1	0.5	1	NA	3.2
**SiS D**	7.25	7.30	1.45	NA	1	0.5	1	NA	0.8

(1)DOTAP: 1,2-Dioleoyl-3-trimethylammonium propane
(2)DOPE: Dioleoyl-phosphatidyl-ethanolamine
(3)mPEG2000-DSPE: N-(carbonyl-methoxypolyethylene glycol-2000)-1,2-distearoyl-sn-glycero-3-phosphoethanolamine, sodium salt
(4)DPPC: Dipalmitoyl-phosphatidyl-choline
(5)PEI: Polyethylenimine
(6)NA: Not applicable

Table S2. Primer sequences and PCR conditions.

Primer pairs	Sequence	PCR conditions
		95° 10’<br>40 cycles 95° 30’’, 60° 30’’, 72° 30’’
$Clcn7^{G213R}$	Fw: CAAGTGCTTCCTCAATA<br>Rv: GCCCTCTTCCAAGCTAAACA	
Il-6	Fw: GTTCTCTGGGAAATCGTGGA<br>Rv: GGAAATTGGGGTAGGAAGGA	
Il-1β	Fw: GCCCATCCTCTGTGACTCAT<br>Rv: AGGCCACAGGTATTTTGTCG	
Tnf-α	Fw: CTCCCTTTGCAGAACTCAGG<br>Rv: AGCCCCCAGTCTGTATCCTT	
Ifn-ɣ	Fw: TTTGAGGTCAACAACCCACA<br>Rv: CGCAATCACAGTCTTGGCTA	
Gapdh	Fw: TGGCAAAGTGGAGATTGTTGC<br>Rv: AAGATGGTGATGGGCTTCCCG	
Clcn3	Fw: GGTCAGGATGGCTTGTTGTT<br>Rv: ACTCTGCCCATGTTTTCCAC	
Clcn5	Fw: GCCTTTGAAGCCACAGAAAG<br>Rv: CTAGCCACCGACTTTTGCTC	

Table S3. Statistical comparison. Data reported in Figure 1 related to the effects induced by the siRNA-SiS12 and siRNA-SiS13 formulations on serum CTX levels (Figure 1B,C) and *$Clcn7^{G213R}$* mRNA expression in femurs (Figure 1D,E) and PBMCs (Figure 1F,G) of treated ADO2 mice were subjected to statistical comparison.

	CTX Fold changes			Clcn7G213R mRNA (Rel. Expression)		
	SiS12-siRNA	Sis13+siRNA	p-value	-		p-value
**Serum**	1.14±0.15	1.18±0,12	ns(1)	-		
**Femurs**	-		-	1.06±0.38	1.00±0.48	ns
**PBMCs**	-		-	0.61±0.18	1.00±0.26	0.024

Statistics: Data are the mean ± SD of n=5 mice per group. Student’s *t* test. (1)ns: statistically not significant.

Table S4. siGLO transfection of AD293 cells.

	siGLO fluorescence							
**Samples**	**Freshly prepared**		**24 h pre-incubation at 4°C**		**24 h pre-incubation at RT(1)**		**24 h pre-incubation at 37°C**	
	**Mean±SD**	**P value**	**Mean±SD**	**P value**	**Mean±SD**	**P value**	**Mean±SD**	**P value**
**Naked siGLO**	31.4±149.0	<0.0001	27.1±50.7	<0.0001	64.5±180.7	<0.0001	122.2±251.8	<0.0001
**siGLO+SiS12**	5984.8±475.6	<0.0001	5877.8±492.2	<0.0001	5083.6±1537.0	<0.01	5036.0±1513.2	<0.0001
**siGLO+LNP**	6254.8±847.6	<0.0001	8315.2±1128.8	<0.0001	5827.4±1740.3	<0.0001	3545.6±170.8	<0.05
**siGLO+LPF complex**	1611.8±374.6	<0.0001	1673.8±250.6	<0.0001	2232.7±437.9	<0.0001	1725.8±332.5	<0.0001
**siGLO+fresh LPF(2)**	4121.8±183.5	ns(4)	5968.0±1845.3	ns	6210.7±884.2	ns	4121.8±183.5	ns
**Untreated(3)**	0.0±79.3	<0.0001	0.0±90.8	<0.0001	0.0±73.7	<0.0001	0.0±49.5	<0.0001
	**Relative siGLO/Hoechst fluorescence**							
**Naked siGLO**	0.003±0.003	<0.0001	0.002±0.004	<0.0001	0.003±0.009	<0.0001	0.009±0.018	<0.0001
**siGLO+SiS12**	0.367±0.367	<0.0001	0.370±0.065	ns	0.480±0.154	<0.0001	0.454±0.089	<0.0001
**siGLO+LNP**	0.214±0.214	<0.0001	0.622±0.091	<0.0001	0.683±0.190	<0.0001	0.216±0.016	<0.0001
**siGLO+LPF complex**	0.094±0.094	<0.0001	0.114±0.023	<0.0001	0.119±0.029	<0.0001	0.117±0.011	<0.0001
**siGLO+fresh LPF(2)**	0.524±0.524	ns	0.405±0.029	ns	0.313±0.030	ns	0.524±0.085	ns
**Untreated(3)**	0.000±0.000	<0.0001	-0.001±0.006	<0.0001	0.000±0.007	<0.0001	0.000±0.003	<0.0001
**Naked siGLO**	0.003±0.003	<0.0001	0.002±0.004	<0.0001	0.003±0.009	<0.0001	0.009±0.018	<0.0001

(1)RT: Room temperature; (2)Positive control; (3)Negative control; (4)ns: Not significant

Note: Ordinary One Way ANOVA with Tukey's multiple comparison test – tabular significance reported for all groups with respect to siGLO+LPF fresh, per each single storage condition tested.

For siGLO fluorescence: On freshly prepared samples: siGLO+SiS12 vs siGLO+LNP (p=0.9980) and Naked siGLO vs Untreated (p=0.2447) were not significant. On 24h pre-incubation at 4°C: Naked siGLO vs Untreated (p=0.9988) was not significant. On 24h pre-incubation at RT: Naked siGLO vs Untreated (p=0.9988) was not significant; siGLO+SiS12 vs siGLO+LNP (p=0.1140) and siGLO+LNP vs. siGLO+fresh LPF (p=0.7706) were not significant. On 24h pre-incubation at 37 °C: Naked siGLO vs Untreated (p=0.9846) was not significant.

For Relative siGLO/Hoechst fluorescence: On freshly prepared samples: siGLO+SiS12 vs siGLO+LNP (p=0.1099) and Naked siGLO vs Untreated (p=0.9999) were not significant. On 24h pre-incubation at 4°C: Naked siGLO vs Untreated (p=0.9999) was not significant. On 24h pre-incubation at RT: Naked siGLO vs Untreated (p=0.9988) was not significant; naked siGLO vs. siGLO+LPF complex reported significance (p=0.0013); siGLO+LPF complex vs. untreated reported significance (p<0.001). On 24h pre-incubation at 37°C: Naked siGLO vs Untreated (p=0.9909) was not significant.

Table S5. Percent of organ SiS12-18:1PE-CF10%+siRNA uptake in vivo.

	Liver	Bones	Kidneys	Lungs	Spleen	Serum
**IP injection**	**% SiS12-18:1PE-CF10%+siRNA RFU (485/Em5-20)**					
**6h**	0	0	1.32	0	1.7	96.98
**24h**	51.12	15.11	13.78	8.51	5.79	5.69
**48h**	56.73	13.52	15.15	7.73	6.87	0
**SC injection**	**% SiS12-18:1PE-CF10%+siRNA RFU (485/Em5-20)**					
**6h**	0	0	0	0	0	100
**24h**	48.02	17.65	16.6	7.89	7.03	2.82
**48h**	47.55	19.45	12.3	14.02	6.67	0

Table S6. Schematic representation of *in vivo* long-term experiment

**Treatment**	**Dose (mg/Kg)**	**N. of mice**
SiS12 combined with naked siRNA $CLCN7^{G215R}$	4	5
SiS12 combined with naked siRNA $CLCN7^{G215R}$	2	5
SiS12 combined with naked siRNA $CLCN7^{G215R}$	0.2	5
SiS12 Empty	SiS12 only	5
Naked siRNA $CLCN7^{G215R}$ alone	4	5
Positive control, generated by combining in-vivoJetPEI® with dAdT siRNA $CLCN7^{G215R}$ (1)	4	5
Untreated wildtype mice injected with saline (NaCl 0.9%)	0.9% NaCl	5

Table S7. ADO2 siRNA sequences

**Length**	**Strand**	**siRNA ADO2 with dTdT overhang**	**siRNA ADO2 with dAdT overhang**
21-mers	Sense	5' AACAGGGUGAAGAUCCCCCdTdT 3'	5' AACAGGGUGAAGAUCCCCCdAdT 3'
21-mers	Antisense	5'GGGGGAUCUUCACCCUGUUdTdT 3'	5' GGGGGAUCUUCACCCUGUUdAdT 3'

Table S8. Animal methods and Animal Research Reporting of In Vivo Experiments (ARRIVE) compliance.

Ethical statement	All the in vivo experiments were conducted in agreement with the national and international guidelines and policies (European Economic Community Council Directive 86/609, OJ L 358, 1, December 12, 1987; Italian Legislative Decree 4.03.2014, n.26, ***Gazzetta Ufficiale della Repubblica Italiana*** no. 61, March 4, 2014) and were approved by the Italian Ministry of Health (approval n. 112/2020-PR).
Study design	Intraperitoneal (i.p.) injections of SiSaf-SiS sshLNP + *Clcn7*^{G213R}-siRNA or SiSaf-SiS sshLNP Empty or Saline in vivo:<br>a. Experimental groups: WT Saline (5 mice), ADO2 SiSaf-SiS sshLNP + *Clcn7*^{G213R}-siRNA (5 mice), ADO2 SiSaf-SiS sshLNP Empty (5 mice).<br>b. Experimental units:<br>i. Young mice (10 days old): groups of animals from different litters.<br>c. Samples that were not analyzed were stored and will be used for other projects, according to the 3Rs principles (reduce).
Experimental procedures	a. siRNA treatment: Mice were injected i.p.. with 0.2, 2 and/or 4 mg/kg of SiSaf-SiS sshLNP + *Clcn7*^{G215R}-siRNA or with SiS sshLNP Empty, 3 times a week for 2 or 4. For pilot experiments by i.p. administration mice were injected once with 4 mg/kg of SiSaf-SiS sshLNP + *Clcn7*^{G215R}-siRNA or with SiS sshLNP Empty and evaluated 48 hours thereafter. Treatments were done in the morning and ended before noon. Animals were monitored for further 3 hours.<br>b. Anesthesia: Ketamine/xylazine cocktail (87.5 mg/kg ketamine, 12.5 mg/kg xylazine) ophthalmic ointment was applied to both eyes to prevent desiccation. To recover from anesthesia mice were placed in warm, clean, dry, quiet environment away from other animals. Commercially available surgical heating pad were used to warm up animals. Bedding material was replaced with toweling material to avoid bedding to stick to eyes or be inhaled while animals were recovering from anesthesia.<br>c. Euthanasia: At the end of the experiments, mice were euthanized by CO_2 inhalation.
Experimental animals	Mice used for in vivo experiments were from the C57BL6/j strain, male, age of 10 days, weight 7± 3gr.<br>Genotype: WT and ADO2.<br>Immunocompetent.
Housing and husbandry	Animal facility: standard.<br>Temperature 20-24°C.<br>Diet: access to food and water *ad libitum*, normal diet (Mucedola code: 3KE25).<br>Dark/light cycle: 12/12 hours.<br>Humidity: 60 ± 5%<br>Cage: plastic.<br>Cage companions, 3 adults/cage, genders were not mixed.<br>Bedding material: high adsorbing power, without dust, changed every week.<br>Environmental enrichment was done with sterile material.
Sample size	Five-5 animals/group were used for the experiments.<br>The sample size was calculated for all the experiments using dedicated software (SigmaPlot v12), based on the expected differences.<br>The efficacy of the siRNA treatment allowed us to obtain significant results with relatively little sample sizes, according to the 3Rs principles.
Allocating animals to experimental groups	Animals were assigned to groups after randomization.

Experimental outcomes	To perform a systematic study to test the likelihood that the therapy could progress towards clinical trials.
Statistical	Statistical analysis was performed by the Student's *t*-test and Multiple Comparison one-way ANalysis Of VAriance (MC ANOVA) according to the type of data sets. The statistical methods are indicated in the figure and table legends. The p-values are indicated in the figures. A p value <0.05 was conventionally considered statistically significant.

Reference

1. Maurizi, A., Capulli, M., Patel, R., Curle, A., Rucci, N., Teti A. (2018). RNA interference therapy for autosomal dominant osteopetrosis type 2. Towards the preclinical development. Bone. *110*, 343-354. https//doi.org/10.1016/j.bone.2018.02.031.
